# Associations of Cholesteryl Ester Transfer Protein TaqIB Polymorphism with the Composite Ischemic Cardiovascular Disease Risk and HDL-C Concentrations: A Meta-Analysis

**DOI:** 10.3390/ijerph13090882

**Published:** 2016-09-05

**Authors:** Shu-xia Guo, Ming-hong Yao, Yu-song Ding, Jing-yu Zhang, Yi-zhong Yan, Jia-ming Liu, Mei Zhang, Dong-sheng Rui, Qiang Niu, Jia He, Heng Guo, Ru-lin Ma

**Affiliations:** 1 Department of Preventive Medicine, Shihezi University School of Medicine, Shihezi 832000, Xinjiang Uyghur Autonomous Region, China; pge888@sina.com (S.-X.G.); ymhldjxa@sina.com (M.-H.Y); 13399931625@163.com (Y.-S.D.); yfyxxzjy@126.com (J.-Y.Z.); erniu19880215@sina.com (Y.-Z.Y.); liujiaming@shzu.edu.cn (J.-M.L.); zmberry@foxmail.com (M.Z.); ruidongsheng@gmail.com (D.-S.R.); niuqiang1214@163.com (Q.N.); hejia123.shihezi@163.com (J.H.); guoheng@shzu.edu.cn (H.G.); 2 Department of Pathology and Key Laboratory of Xinjiang Endemic and Ethnic Diseases (a Joint Venture with the Chinese Ministry of Education), Shihezi University School of Medicine, Shihezi 832000, Xinjiang Uyghur Autonomous Region, China

**Keywords:** cholesteryl ester transfer protein, polymorphism, composite ischemic cardiovascular disease, HDL-C, meta-analysis

## Abstract

*Background*: Previous studies have evaluated the associations between the cholesteryl ester transfer protein (CETP) *TaqIB* polymorphism (rs708272), the risk of developing composite ischemic cardiovascular disease (CVD) and the concentration of high-density lipoprotein cholesterol (HDL-C), but results remain controversial. The objective of this study was to investigate whether a relationship exists between these factors. *Methods*: We conducted a meta-analysis of available studies to clarify the associations of the *CETP TaqIB* polymorphism with HDL-C concentration and the composite ischemic CVD risk in both Asians and Caucasians. All statistical analyses were done with Stata 12.0. *Results*: Through utilization of the Cochrane Library, Embase, PubMed, Web of Science, Springer, China Science and Technology Journal Database, China National Knowledge Infrastructure, Google Scholar, and Baidu Library, a total of 45 studies from 44 papers with 20,866 cases and 21,298 controls were combined showing a significant association between the *CETP TaqIB* variant and composite ischemic CVD risk. Carriers of allele *TaqIB-B1* were found to have a higher risk of composite ischemic CVD than non-carriers: OR = 1.15, 95% CI = 1.09–1.21, *p* < 0.001. Meanwhile, 28 studies with 23,959 subjects were included in the association between the *CETP TaqIB* polymorphism and the concentration of HDL-C. Results suggested that carriers of the *B1B1* genotype had lower concentrations of HDL-C than those of the *B2B2* genotype: SMD = 0.50, 95% CI = 0.36–0.65, *p* < 0.001. *Conclusions*: The synthesis of available evidence demonstrates that the *CETP TaqIB* polymorphism protects against composite ischemic CVD risk and is associated with a higher HDL-C concentration in both Asians and Caucasians.

## 1. Introduction

Composite ischemic cardiovascular disease (CVD), including coronary artery disease (CAD), ischemic stroke (IS), and myocardial infarction (MI) has become a serious public health problem around the world because of their high morbidity and mortality [1,2]. However, their exact mechanisms are still unclear. For a long time, atherosclerosis (AS) has attracted attention because it is the pathological foundation of CAD, IS, and MI. Abnormal cholesterol metabolism was considered to be the main factor for atherosclerosis, and epidemiological evidence considered low concentrations of serum high-density lipoprotein cholesterol (HDL-C) to be an independent risk factor [3,4]. However, high-density lipoprotein (HDL) has now been shown to play a pivotal role in mediating the transfer of cholesterol from extra hepatic tissues to the liver and reducing the deposition of cholesterol on the artery wall [5].

Serum HDL-C concentrations are affected by many genetic and environmental factors. The cholesteryl ester transfer protein (CETP) gene located on chromosome 16q21, encodes the key plasma protein that mediates the transfer of esterified cholesterol from HDL to apolipoprotein B-containing particles in exchange for triglycerides [6,7]. Mutation of the gene may affect the transcription and expression of CETP, thereby affecting serum HDL-C concentrations [8]. The *CETP TaqIB* (rs708272) polymorphism is the most common polymorphism in intron 1 of the *CETP* gene and its mutation can affect the concentration as well as activity of plasma CETP, which affected the level of HDL-C [9]. Recently, though numerous studies have shown a relationship between the *CETP TaqIB* polymorphism in the synthesis of HDL-C and composite ischemic CVD risk, research has remained inconsistent, possibly due to the small sample sizes used in the individual studies.

In 2005, Boekholdt et al. performed a meta-analysis to evaluate the association the *CETP TaqIB* polymorphism in the synthesis of serum HDL-C and CAD risk, and demonstrated that the *CETP TaqIB* variant is associated with HDL-C level and CAD risk in Caucasians [10]. Li et al. also conducted a meta-analysis to evaluate the association of this variant with CAD in Chinese; however, no relationship between the *CETP TaqIB* polymorphism and CAD was observed [11]. Cao et al. and Wang et al. performed meta-analysis to evaluate the association the *CETP TaqIB* variant and MI. Their results showed that the *CETP TaqIB-B2* allele protects against the development of MI [12,13]. No meta-analysis was found on the association between the *CETP TaqIB* polymorphism and IS. Considering the four meta-analyses above focused only on the association of the *CETP TaqIB* polymorphism with a single atherosclerotic disease and results were controversial in regards to ethnicity (Asians and Caucasians), we performed this meta-analysis to clarify the role of the *CETP TaqIB* polymorphism in the synthesis of HDL-C and the composite ischemic CVD risk.

## 2. Materials and Methods

### 2.1. Literature Search

The protocol was approved by the Institutional Ethics Review Board (IERB) of the First Affiliated Hospital of Shihezi University School of Medicine (IERB No. SHZ2010LL01). Using the standards of the Meta-analysis of Observational Studies in Epidemiology group (MOOSE) [14] and the Preferred Reporting Items for Systematic Reviews and Meta-analyses (PRISMA) [15], searches were performed using the following electronic databases: the Cochrane Library, Embase, PubMed, Web of Science, Springer, China Science and Technology Journal Database (CSTJ), China National Knowledge Infrastructure (CNKI), Google Scholar, and Baidu Library (the last search was conducted on 31 January 2016). Searches were performed using combinations of the following key words: (“cholesteryl ester transfer protein” OR “CETP”) and (“variation” OR “variant” OR “mutation” OR “polymorphism” OR “genotype”) and (“CAD” OR “coronary artery disease” OR “coronary heart disease” OR “CHD” OR “myocardial infarction” OR “MI” OR “ischemic cardiovascular disease” OR “IS”) and (“high-density lipoprotein cholesterol” OR “HDL-C” OR “blood lipid” OR “serum lipid”).

### 2.2. Eligibility Criteria

The eligibility criteria for the inclusion of articles in the present meta-analysis were the following: (1) The publication evaluated the associations of the *CETP TaqIB* polymorphism with AS or HDL-C level; (2) CAD and MI diagnosis required the result of coronary angiography, and the diagnosis of IS depended on the result of magnetic resonance imaging or computed tomography; (3) published in either Chinese or English; (4) for the composite ischemic CVD association, sufficient published data for calculating odds ratios (ORs) with their 95% confidence intervals (CIs); for HDL-C concentrations association, the population, the mean of HDL-C concentrations, and the standard deviations (SD) by genotype should be available.

### 2.3. Exclusion Criteria

The exclusion criteria were as follows: (1) Duplicate publications; (2) incomplete information; (3) insufficient or insignificant statistical data; (4) review articles.

### 2.4. Data Extraction

Two reviewers (Minghong Yao and Yusong Ding) independently screened full-length articles according to the pre-specified inclusion criteria. For the composite ischemic CVD association, the following information was extracted: name of the first author, year of publication, study population (country, ethnicity), source of controls, case/control sample size, minor allele frequency (MAF), genotype counts in the cases/controls, and evidence of Hardy-Weinberg equilibrium (HWE); for HDL-C concentrations association, name of the first author, year of publication, study population (country, ethnicity), population number, mean of HDL-C concentrations, and their SD by genotype. If key data were not presented in the relevant publications, we tried to obtain them directly from the authors of the relevant studies. When the two reviewers’ opinions differed, a third reviewer (ShuXia Guo) was asked to make final decisions regarding the results.

### 2.5. Quality Assessment for Individual Studies

The Newcastle-Ottawa Scale (NOS) was used to assessed the methodologic quality of the individual studies by two reviewers (Minghong Yao and Yizhong Yan) [16]. Each study was evaluated and scored based on three criteria: selection (4 stars), comparability (2 stars), and exposure (3 stars). The NOS point ranges between zero up to nine stars. Any disagreement was resolved by discussion with a third reviewer (Jiaming Liu).

### 2.6. Data Analysis

All statistics were analyzed in Stata 12.0 (StataCorp, College Station, TX, USA). All the tests were two-sided and a *p*-value of less than 0.05 was considered statistically significant. The HWE was assessed using the chi-square test. The strength of associations between the *CETP TaqIB* polymorphism and atherosclerosis were assessed by summary odds ratios (ORs) with 95% confidence intervals (CIs). Pooled ORs were performed for the allele contrasts as followed: (*B1* allele vs. *B2* allele), additive genetic model (*B1B1* vs. *B2B2*), recessive genetic model (*B1B1* vs. *B1B2* + *B2B2*), and dominant genetic model (*B1B1* + *B1B2* vs. *B2B2*), respectively. A pooled standardized mean difference (SMDs) and its 95% CIs were used for the meta-analysis of HDL-C concentrations and the *CETP TaqIB* polymorphism. Heterogeneity across individual studies was calculated using the Cochran’s-*Q* statistic and the I^2^ statistic (*p* < 0.10 and I^2^ > 50% indicated evidence of heterogeneity) [17,18]. With no heterogeneity among studies, the ORs or SMDs estimate of each study was calculated by the fixed effect model (Mantel-Haenszel) [19]. Otherwise, the random effect model (DerSimonian and Laird) was used [20,21]. Subsequently, the Galbraith plot and meta-regression were performed to explore the sources of heterogeneity [22]. For the composite ischemic CVD association, subgroup analyses were performed based on ethnicity, atherosclerotic diseases, source of controls, and study type; for HDL-C association, subgroup analyses were performed based on ethnicity. Sensitivity analyses were performed based on HWE (studies without HWE were excluded) and sample size (*n* < 400 were excluded). Potential risk of publication bias was tested by funnel plot and Egger’s test.

## 3. Results

### 3.1. Selection and Characteristics of Studies

**Figure 1:**
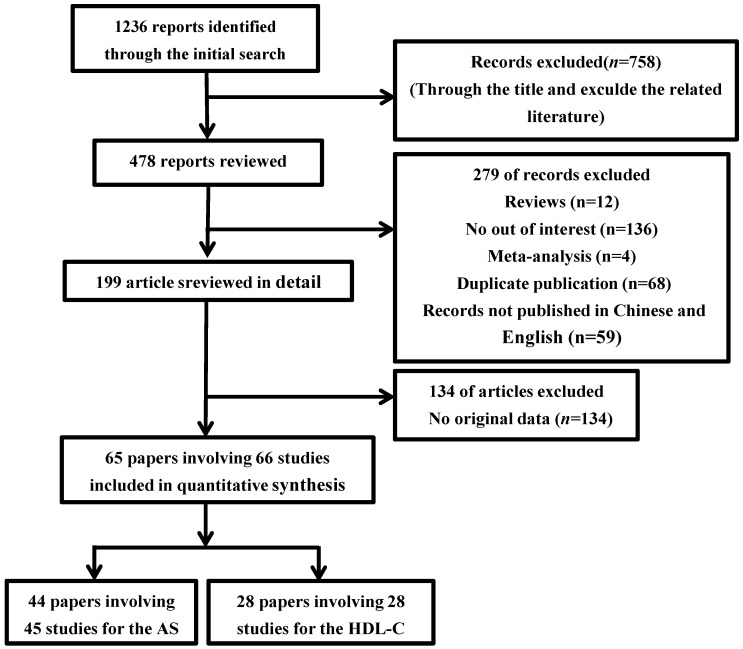
Flow diagram of the study selection process.

The present study met the PRISMA statements (Checklist S1) and MOOSE guidelines (Table S1). The study selection process is detailed in Figure 1. Through a comprehensive retrieval and evaluation, 45 studies from 44 papers with 20,866 cases and 21,298 controls met the inclusion criteria to assess the association between the *CETP* *TaqIB* polymorphism and the composite ischemic CVD [23,24,25,26,27,28,29,30,31,32,33,34,35,36,37,38,39,40,41,42,43,44,45,46,47,48,49,50,51,52,53,54,55,56,57,58,59,60,61,62,63,64,65]. The selected study characteristics and data are listed in Table 1. Among these studies, 28 involved CAD [23,24,25,26,27,28,29,30,31,32,34,35,36,37,38,39,44,46,47,50,52,53,54,55,59,60,61,66], three involved IS [63,64,65], and 14 involved MI [33,40,41,42,43,45,48,49,51,56,57,58,62]. In addition, there were 26 studies on Caucasians [23,24,25,27,30,38,39,40,41,42,43,44,45,47,48,50,51,53,56,57,58,60,62,63,64] and 19 studies on Asians [26,28,29,31,32,33,34,35,36,37,46,49,52,54,55,59,61,65,66]. Controls of 23 studies were hospital-based [23,24,25,26,27,28,29,30,31,32,33,34,35,36,37,57,58,59,61,63,64,65,66], while those of the other 22 studies were population-based [38,39,40,41,42,43,44,45,46,47,48,49,50,51,52,53,54,55,56,60,62]. Seven studies did not follow the Hardy-Weinberg equilibrium [23,35,36,40,42,43,58]. In addition, NOS results showed that the average scores were 6.8.

Table 2 describes the characteristics of studies included in the association between the *CETP TaqIB* polymorphism and serum HDL-C concentrations. A total of 28 studies with 23,959 subjects were included in the analysis [8,33,35,36,40,44,45,50,53,59,67,68,69,70,71,72,73,74,75,76,77,78,79,80,81,82,83,84,85]. Of these, there were 11 studies on Caucasians [8,40,44,45,50,53,67,69,71,81,83] and 17 studies on Asians [33,35,36,59,68,70,72,73,74,75,76,77,78,79,80,82,84,85]. Five studies did not follow the HWE [35,72,74,76,77]. Additionally, NOS results showed that the average scores were 6.4.

### 3.2. Association between the CETP TaqIB Polymorphism and the Composite Ischemic CVD Risk

**Figure 2:**
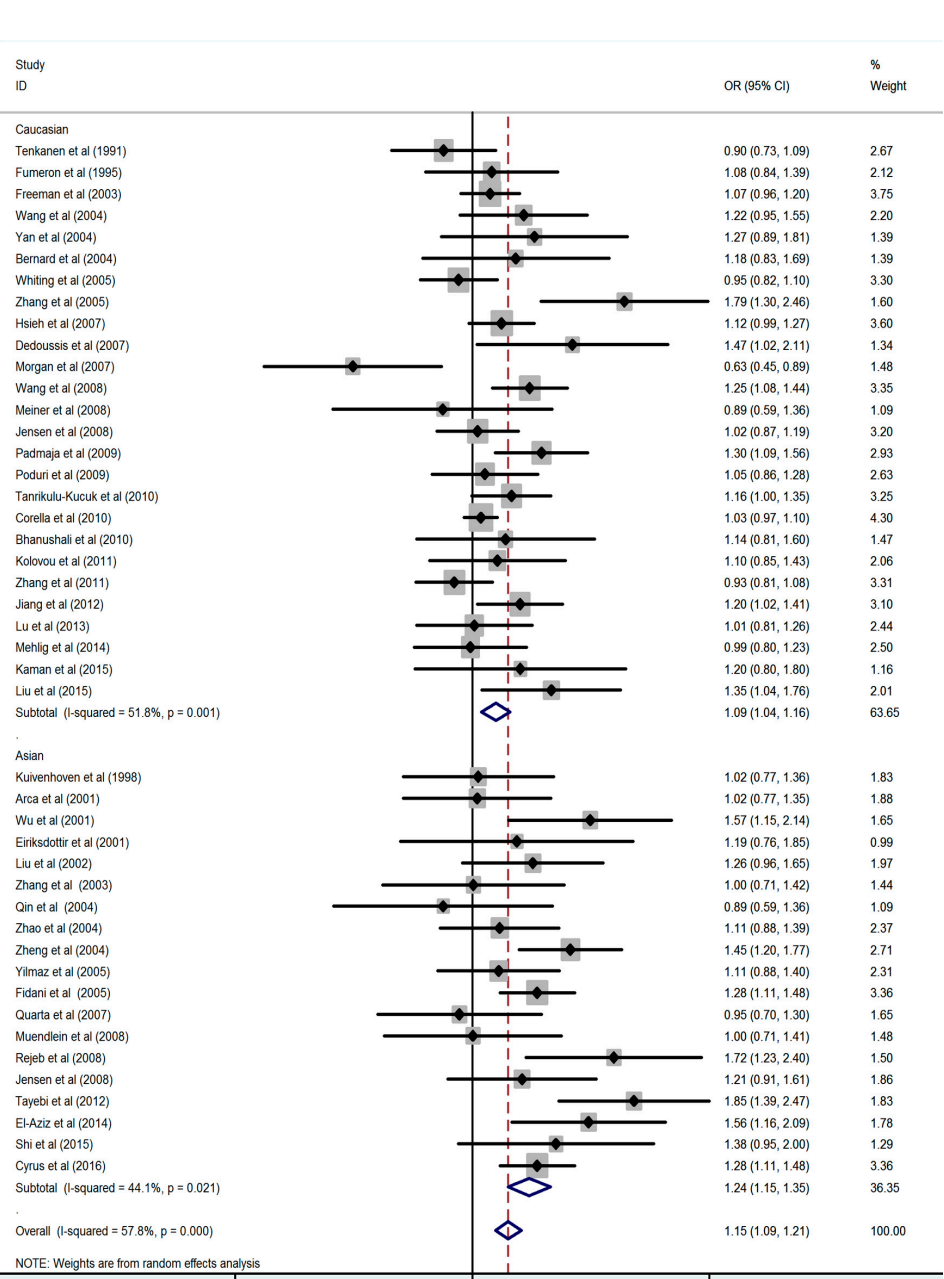
Meta-analysis of atherosclerosis and the *CETP TaqIB* polymorphism (*B1* vs. *B2*).

The results of all 45 comparisons showed evidence of a significant association between the *CETP TaqIB* polymorphism and the composite ischemic CVD, suggesting that carriers of allele *TaqIB*-*B1* have a higher risk of the composite ischemic CVD than non-carriers (OR = 1.15, 95% CI = 1.09–1.21) (Figure 2). The additive genetic model (*B1B1* vs. *B2B2*: OR = 1.26, 95% CI = 1.19–1.34), dominant genetic model (*B1B1* + *B1B2* vs. *B2B2*: OR = 1.20, 95% CI = 1.14–1.27), and recessive genetic model (*B1B1* vs. *B1B2* + *B2B2*: OR = 1.13, 95% CI = 1.08–1.18) were also included in the analysis and results were similar with allele comparison (Figures S1–S3). Subgroup analyses by ethnicity showed significant associations in Asians consistent with that in Caucasians. In addition, significant associations were also found between this variant and susceptibility to the composite ischemic CVD in the population-based group, the hospital-based group, the CAD group, the MI group, the IS group, the case control study group, and the cohort study group, respectively. We also observed the association between *CETP TaqIB*-*B2* polymorphism and the composite ischemic CVD risk where was stronger in the Asian than the Caucasians. The main results of the meta-analysis are shown in Table 3.

### 3.3. Association between the CETP TaqIB Polymorphism and HDL-C Concentrations

**Figure 3:**
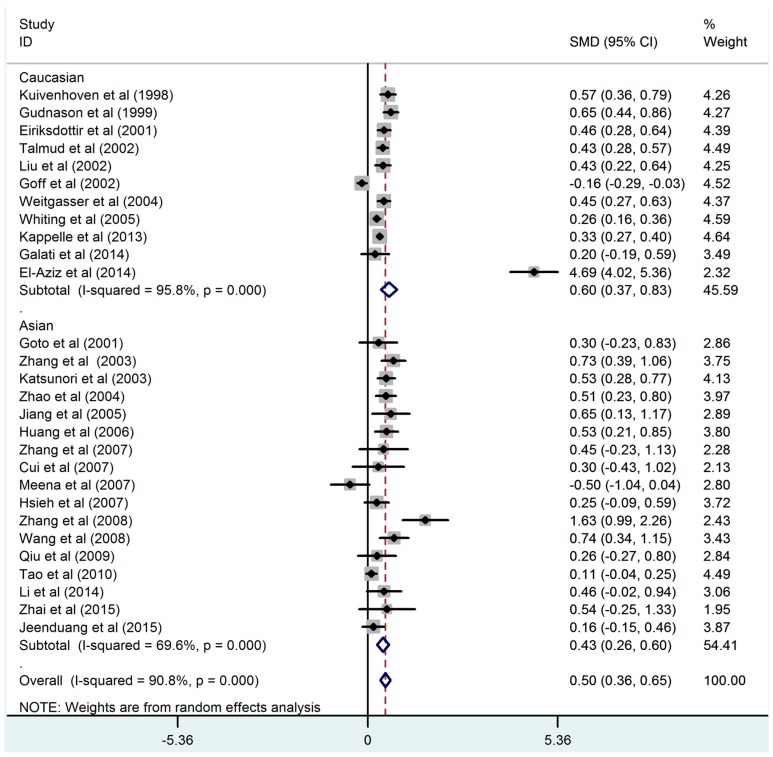
Association between the *CETP TaqIB* polymorphism and HDL-C level (*B1B1* vs. *B2B2*).

Figure 3 describes the results of the meta-analysis of the *CETP TaqIB* polymorphism and HDL-C concentrations. Our analysis strongly suggested that carriers of the *B1B1* genotype had lower concentrations of HDL-C than those of the *B2B2* genotype (*B1B1* vs. *B2B2*: SMD = 0.50, 95% CI = 0.36–0.65). We also compared carriers of the *B1B1* genotype with those of the *B1B2* genotype (Figure S4: *B1B1* vs. *B1B2*: SMD = 0.18, 95% CI = 0.10–0.26) and *B1B2* genotype with those of *B2B2* genotype (Figure S5: *B1B2* vs. *B2B2*: SMD = 0.32, 95% CI = 0.21–0.42). Subgroup analyses by ethnicity confirmed that the relationship between the *CETP TaqIB*-*B2* polymorphism and the HDL-C concentration in Asians was less consistent than that in Caucasians (Figure 2, Figures S4 and S5).

### 3.4. Sensitivity Analysis

Sensitivity analysis was performed to determine the robustness of the study results. The included studies were limited to those conforming to HWE and sample size. We performed sensitivity analysis by removing studies without HWE and an *n* < 400. Overall, the corresponding pooled ORs and SMD were not materially altered for either analysis. Results of the sensitivity analysis suggested that the overall results were relatively robust and credible. The main results of the sensitivity analyses are shown in Table 3 and Figures S6–S11.

### 3.5. Heterogeneity Analysis

For the relationship between the *CETP TaqIB* polymorphism and the composite ischemic CVD, significant heterogeneity among the available studies were observed in the overall comparisons for the allelic model: *P_Q_* < 0.001, I^2^ = 57.8%; additive model: *P_Q_* < 0.001, I^2^ = 55.8%; recessive model: *P_Q_* < 0.001, I^2^ = 52.0%; and dominant model: *P_Q_* = 0.001, I^2^ = 41.7%. To clarify the sources of heterogeneity, we conducted a meta-regression analysis. The results showed that heterogeneity can be explained by the source of controls for the allelic model: *p* = 0.046, additive model: *p* = 0.025, and dominant model: *p* = 0.039, and ethnicity for the additive model: *p* = 0.048. 

For the relationship between the *CETP TaqIB* polymorphism and HDL-C concentrations, significant heterogeneity among the available studies was also observed in the overall comparisons for *B1B1* vs. *B2B2*: *P_Q_* < 0.001, I^2^ = 90.8%; *B1B1* vs. *B1B2*: *P_Q_* < 0.001, I^2^ = 79.9%; and *B1B2* vs. *B2B2*: *P_Q_* < 0.001, I^2^ = 85.1%. Four studies were identified as the main contributors of heterogeneity in the Asian studies [74,76,77,80] and four studies were identified as the main contributors of heterogeneity in the Caucasian studies [44,50,67,69] using the Galbraith plot (Figures S12 and S13). Figures S14–S16 show the association between the *CETP TaqIB* polymorphism and HDL-C concentrations after exclusion of these outlier studies. However, the significant association between the *CETP* polymorphism and HDL-C concentrations was unchanged both in the Asian subgroup (*B1B1* vs. *B2B2*: SMD = 0.47, 95% CI = 0.36–0.57; *B1B1* vs. *B1B2*: SMD = 0.19, 95% CI = 0.11–0.26; *B1B2* vs. *B2B2*: SMD = 0.28, 95% CI = 0.18–0.37) and Caucasian subgroup (*B1B1* vs. *B2B2*: SMD = 0.35, 95% CI = 0.30–0.40; *B1B1* vs. *B1B2*: SMD = 0.16, 95% CI = 0.12–0.19; *B1B2* vs. *B2B2*: SMD = 0.19, 95% CI = 0.15–0.20).

### 3.6. Publication Bias

**Figure 4:**
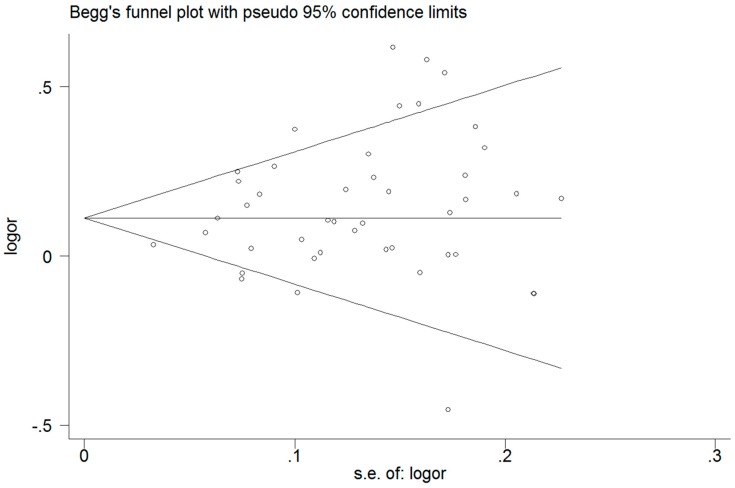
Funnel plot for allele comparison of atherosclerosis and the *CETP TaqIB* polymorphism. Each small circle represents a separate study for the indicated association.

**Figure 5:**
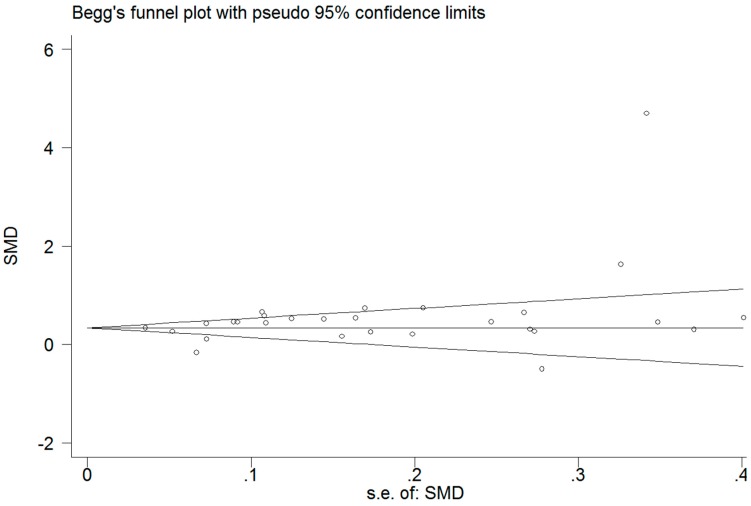
Funnel plot of *CETP TaqIB* polymorphism and HDL-C level (*B1B1* vs. *B2B2*). Each small circle represents a separate study for the indicated association.

Funnel plots and Egger’s test were performed to access the publication bias of literature. For the *CETP* polymorphism and the composite ischemic CVD risk analysis (*B1* vs. *B2*), the shape of the funnel plot (Figure 4) did not reveal obvious asymmetry, which means no publication bias. This was confirmed by Egger’s test (*p* = 0.074). For the *CETP* polymorphism and HDL-C analysis (*B1B1* vs. *B2B2*), neither the shape of the funnel plot (Figure 5) nor Egger’s test (*p* = 0.058) revealed any obvious asymmetry.

## 4. Discussion

In the present meta-analysis, a total of 45 studies from 44 papers with 20,866 cases and 21,298 controls, we found that the *TaqIB*-*B2* allele was significantly associated with reduction of composite ischemic CVD both in Caucasians and Asians. Additionally, 28 studies with 23,959 subjects were included in the analysis on the association between the *CETP TaqIB* polymorphism and HDL-C concentrations. According to the results, the *TaqIB*-*B2* allele was significantly associated with a higher level of HDL-C both in Caucasians and Asians. Therefore, it is reasonable to assume that the *CETP TaqIB* polymorphism is influencing HDL-C metabolism to protect against the development of AS. This result suggests that we can use *CETP* inhibitors to prevent and treat dyslipidemia and the composite ischemic CVD. In 2014, Keene et al. performed a meta-analysis to investigate association between the CETP inhibitors and cardiovascular outcomes [86]. The results show that CETP inhibitors neither increase the serum HDL-C concentration nor reduce the mortality rate of the composite ischemic CVD. It is probably because the trial design or the use of a drug with serious off-target adverse effects. On the other hand, it is well known that the serum HDL-C concentrations affected by multiple environmental and genetic factors. Therefore, the use of CETP inhibitor alone may not be able to reduce the risk of having a clinical atherosclerotic cardiovascular event.

To create a more comprehensive analysis of the association between the *CETP TaqIB* polymorphism and composite ischemic CVD, we performed subgroup analyses based on ethnicity, source of controls, atherosclerotic disease, and study type in the allelic model, additive model, recessive model, and dominant model. Significant associations were found between this variant and susceptibility to composite ischemic CVD in the Caucasian group, Asian group, population-based group, hospital-based group, IS group, CAD group, MI group (except for the recessive model), case control study group, and the subgroup of the cohort study group (except for the recessive model), respectively. For the association between the *CETP TaqIB* polymorphism and HDL-C, we also performed subgroup analysis based on ethnicity in the *B1B1* vs. *B2B2* model, *B1B2* vs. *B2B2* model, and *B1B1* vs. *B1B2* model. Significant associations were found between this variant and serum HDL-C concentrations in both the Caucasian and Asian group. These results further strengthen the conclusion that the *CETP* *TaqIB*-*B2* allele protects against atherosclerosis by influencing HDL-C metabolism both in Asians and Caucasians. We also found that the association between *CETP TaqIB*-*B2* polymorphism and composite ischemic CVD risk was stronger in Asians than Caucasians, but the relationship between the *CETP TaqIB-B2* polymorphism and the HDL-C concentration in Asians was less consistent than that in Caucasians, which can be attributed to different environmental factors, lifestyle, etc.

Considering the influence of small-study effects on the overall results, we performed sensitivity analyses by excluding studies with low sample size or without the HWE. However, the corresponding pooled ORs and SMDs were unchanged in all comparisons, indicating statistically robust results.

Meanwhile, the existence of heterogeneity among the available studies, either for the *CETP TaqIB* polymorphism and composite ischemic CVD or for the *CETP TaqIB* polymorphism and HDL-C may affect the reliability of the results to a large extent. For the relationship between *CETP TaqIB* polymorphism and composite ischemic CVD, the heterogeneity can be explained by the source of controls (hospital controls and population controls) and ethnicity (Asians and Caucasians); for the relationship between *CETP TaqIB* polymorphism and serum HDL-C concentrations, the Galbraith plot was used to detect the source of heterogeneity for Asians and Caucasians. We identified four studies were as the main contributors of heterogeneity for Asians [74,76,77,80] and four for Caucasians [44,50,67,69]. The heterogeneity among Asians and Caucasians was effectively removed after excluding these outliers; however, the significant association between the *CETP TaqIB* polymorphism and serum HDL-C concentrations was unchanged. According to these outlier studies, the heterogeneity may be explained by the HWE, sample size, and disease.

There are several potential limitations in our present meta-analysis that should be acknowledged. First, there was significant heterogeneity in our study. Although we used appropriate meta-analytic techniques, we could not completely exclude the influence of the heterogeneity. Second, we may have missed eligible articles reported in other languages because our study only focused on articles published in English and Chinese. Third, the sample sizes of some studies were rather small. In summary, it is well-known that the composite ischemic CVD is affected by multiple environmental and genetic factors. Here, we discussed a single gene polymorphism and its impact on disease; however, several factors remain to be elucidated.

## 5. Conclusions

The present meta-analysis shows that the *CETP TaqIB*-*B2* allele is associated with a higher serum HDL-C concentration and plays a protective role in composite ischemic CVD risk both in Asians and in Caucasians. Further investigations with the consideration of genetic and environmental interactions are needed.

## Figures and Tables

**Table 1:** Characteristics of individual studies included in the meta-analysis of atherosclerosis and the *CETP TaqIB* polymorphism.

First Author	Year	Country	Ethnicity	Disease	Source of Controls	Study Type	Size (Case/Control)	MAF	HWE	Genotypes Distribution (Case/Control)			Score
										*B1B1*	*B1B2*	*B2B2*	
Tenkanen et al. [51]	1991	Finland	Caucasian	MI	PB	CS	72/115	0.44	Yes	19/33	40/65	13/17	8
Fumeron et al. [41]	1995	France	Caucasian	MI	PB	CCS	608/724	0.40	Yes	209/258	312/346	87/120	8
Kuivenhoven et al. [44]	1998	The Netherlands	Caucasian	CAD	PB	CS	380/427	0.41	Yes	129/152	183/214	68/61	7
Wu et al. [33]	2001	China	Asian	MI	HB	CCS	149/274	0.46	Yes	45/63	79/159	25/52	8
Arca et al. [38]	2001	Italy	Caucasian	CAD	PB	CCS	408/180	0.41	Yes	153/67	187/77	68/36	8
Eiriksdottir et al. [40]	2001	Iceland	Caucasian	MI	PB	CS	378/745	0.45	No	128/194	191/396	59/155	8
Liu et al. [45]	2002	USA	Caucasian	MI	PB	CS	384/384	0.43	Yes	125/122	196/193	63/69	8
Freeman et al. [56]	2003	UK	Caucasian	MI	PB	CS	499/1105	0.50	Yes	164/239	259/541	76/225	8
Zhang et al. [35]	2003	China	Asian	CAD	HB	CCS	234/164	0.41	No	76/49	126/95	32/20	6
Qin et al. [29]	2004	China	Asian	CAD	HB	CCS	249/167	0.41	Yes	81/49	131/97	37/21	6
Wang et al. [32]	2004	China	Asian	CAD	HB	CCS	128/247	0.42	Yes	50/72	66/123	12/52	6
Yan et al. [34]	2004	China	Asian	CAD	HB	CCS	106/64	0.41	Yes	41/19	46/34	19/11	6
Zhao et al. [36]	2004	China	Asian	CAD	HB	CCS	238/203	0.41	No	95/60	105/109	38/34	6
Zheng et al. [37]	2004	China	Asian	CAD	HB	CCS	203/100	0.39	Yes	66/33	114/55	23/12	6
Bernard et al. [43]	2004	UK	Caucasian	MI	PB	CCS	4442/3273	0.43	No	1477/1100	2175/1527	790/646	8
Yilmaz et al. [42]	2005	Turkey	Caucasian	MI	PB	CCS	173/111	0.42	No	66/39	72/46	35/26	6
Fidani et al. [63]	2005	Greek	Caucasian	IS	HB	CCS	96/100	0.41	Yes	35/34	47/45	14/21	6
Whiting et al. [53]	2005	USA	Caucasian	CAD	PB	CS	2392/827	0.42	Yes	792/279	1200/377	400/171	8
Zhang et al. [54]	2005	China	Asian	CAD	PB	CCS	88/94	0.41	Yes	31/32	40/50	17/12	6
Dedoussis et al. [57]	2007	Greece	Caucasian	MI	HB	CCS	237/237	0.41	Yes	83/78	121/120	33/39	7
Morgan et al. [58]	2007	USA	Caucasian	MI	HB	CCS	805/656	0.44	No	250/224	387/297	168/135	6
Hsieh et al. [59]	2007	China	Asian	CAD	HB	CCS	101/264	0.31	Yes	19/23	47/111	35/130	5
Quarta et al. [64]	2007	Italy	Caucasian	IS	HB	CCS	215/236	0.43	Yes	79/73	105/108	31/55	6
Muendlein et al. [27]	2008	Austria	Caucasian	CAD	HB	CS	332/225	0.40	Yes	125/71	162/116	45/38	8
Rejeb et al. [30]	2008	Tunisian	Caucasian	CAD	HB	CS	212/104	0.41	Yes	104/45	93/47	15/12	8
Meiner et al. [48]	2008	USA	Caucasian	MI	PB	CCS	550/620	0.45	Yes	173/166	282/320	95/134	6
Wang et al. [52]	2008	China	Asian	CAD	PB	CCS	317/298	0.41	Yes	117/99	148/146	52/53	6
Jensen et al. [62] ^a^	2008	USA	Caucasian	MI	PB	CS	247/486	0.42	Yes	84/166	120/235	42/85	8
Jensen et al. [62] ^b^	2008	USA	Caucasian	MI	PB	CS	259/513	0.41	Yes	89/180	126/244	44/89	8
Padmaja et al. [28]	2009	Indian	Asian	CAD	HB	CCS	504/338	0.45	Yes	163/86	264/161	77/91	6
Poduri et al. [49]	2009	India	Asian	MI	PB	CCS	265/150	0.41	Yes	117/3	107/82	41/35	6
Tanrikulu-Kucuk et al. [23]	2010	Turkey	Caucasian	CAD	HB	CCS	135/112	0.46	No	40/33	71/50	24/29	6
Corella et al. [39]	2010	Spanish	Caucasian	CAD	PB	CS	557/1180	0.47	Yes	224/482	247/537	86/161	8
Bhanushali et al. [66]	2010	Indian	Asian	CAD	HB	CCS	90/150	0.46	Yes	33/38	40/77	17/35	7
Kolovou et al. [25]	2011	Greek	Caucasian	CAD	HB	CCS	374/96	0.42	Yes	126/22	202/45	46/29	6
Zhang et al. [55]	2011	China	Asian	CAD	PB	CCS	334/301	0.34	Yes	172/136	106/120	56/45	8
Jiang et al. [65]	2012	China	Asian	IS	HB	CCS	220/220	0.29	Yes	130/103	72/86	18/31	6
Tayebi et al. [61]	2012	Singapore	Asian	CAD	HB	CCS	659/927	0.45	Yes	228/245	322/491	109/191	7
Lu et al. [46]	2013	Singapore	Asian	CAD	PB	CCS	659/927	0.45	Yes	228/245	322/491	109/191	8
Mehlig et al. [47]	2014	Sweden	Caucasian	CAD	PB	CCS	618/2921	0.43	Yes	209/938	313/1420	96/563	8
El-Aziz et al. [50]	2014	Egypt	Caucasian	CAD	PB	CCS	116/119	0.46	Yes	38/30	60/57	18/32	6
Kaman et al. [24]	2015	Turkey	Caucasian	CAD	HB	CCS	210/100	0.44	Yes	44/29	81/45	85/26	6
Liu et al. [26]	2015	China	Asian	CAD	HB	CCS	322/108	0.42	Yes	113/40	145/47	64/21	6
Shi et al. [31]	2015	China	Asian	CAD	HB	CCS	312/88	0.42	Yes	112/29	138/44	62/15	6
Cyrus et al. [60]	2016	Saudi Arabia	Caucasian	CAD	PB	CCS	990/618	0.41	Yes	376/183	454/321	160/114	6

a: Nurses’ Health Study, b: Health Professionals Follow-up Study, USA: The United States, UK: United Kingdom, CAD: coronary artery disease, MI: myocardial infraction, IS: ischemic stroke, HB: hospital-based, PB: population-based, MAF: minor allele frequencies, HWE: Hardy-Weinberg equilibrium, CS: cohort study, CCS: case control study.

**Table 2:** Characteristics of individual studies included in the meta-analysis of HDL-C level and the *CETP TaqIB* polymorphism.

First Author	Year	Country	Ethnicity	MAF	HWE	*B1B1*			*B1B2*			*B2B2*			Score
						Mean	SD	*n*	Mean	SD	*n*	Mean	SD	*n*	
Kuivenhoven et al. [44]	1998	The Netherlands	Caucasian	0.41	Yes	0.88	0.21	281	0.93	0.21	397	1.01	0.26	129	7
Gudnason et al. [67]	1999	Mixed	Caucasian	0.44	Yes	1.13	0.21	237	1.19	0.24	380	1.27	0.22	150	7
Eiriksdottir et al. [40]	2001	Iceland	Caucasian	0.45	Yes	1.09	0.31	328	1.12	0.29	596	1.25	0.40	210	8
Goto et al. [68]	2001	Japan	Asian	0.43	Yes	1.14	0.28	37	1.23	0.37	47	1.23	0.33	22	6
Talmud et al. [8]	2002	UK	Caucasian	0.45	Yes	0.79	0.25	500	0.84	0.25	896	0.90	0.27	317	6
Liu et al. [45]	2002	USA	Caucasian	0.43	Yes	1.17	0.28	247	1.24	0.34	389	1.30	0.34	132	8
Goff et al. [69]	2002	UK and France	Caucasian	0.47	Yes	1.33	0.40	410	1.29	0.60	889	1.26	0.45	504	7
Zhang et al. [35]	2003	China	Asian	0.41	No	1.26	0.22	125	1.30	0.25	221	1.42	0.22	52	6
Katsunori et al. [70]	2003	Japan	Asian	0.4	Yes	1.32	0.46	217	1.43	0.57	279	1.59	0.62	95	7
Zhao et al. [36]	2004	China	Asian	0.41	Yes	1.19	0.36	155	1.27	0.34	214	1.38	0.39	72	6
Weitgasser et al. [71]	2004	Austrian	Caucasian	0.41	Yes	1.49	0.39	358	1.55	0.41	475	1.67	0.41	184	7
Jiang et al. [72]	2005	China	Asian	0.37	No	1.16	0.27	49	1.20	0.33	38	1.34	0.29	21	6
Whiting et al. [53]	2005	USA	Caucasian	0.42	Yes	0.91	0.33	1071	0.95	0.34	1577	1.00	0.38	571	8
Huang et al. [73]	2006	China	Asian	0.40	Yes	1.08	0.29	121	1.13	0.29	163	1.27	0.48	56	6
Zhang et al. [74]	2007	China	Asian	0.40	No	1.26	0.31	24	1.34	0.35	20	1.42	0.43	13	6
Cui et al. [75]	2007	China	Asian	0.46	Yes	1.44	0.32	17	1.58	0.46	24	1.54	0.36	13	6
Meena et al. [76]	2007	Indian	Asian	0.21	No	1.20	0.20	15	1.10	0.10	36	1.10	0.20	106	6
Hsieh et al. [59]	2007	China	Asian	0.31	Yes	43.31	10.63	42	43.39	11.09	158	46.24	11.83	165	5
Zhang et al. [77]	2008	China	Asian	0.39	No	1.45	0.31	46	1.41	0.23	78	2.03	0.47	16	6
Wang et al. [78]	2008	China	Asian	0.44	Yes	1.31	0.38	66	1.39	0.38	98	1.61	0.44	41	6
Qiu et al. [79]	2009	China	Asian	0.41	Yes	1.18	0.36	38	1.25	0.33	32	1.28	0.42	21	6
Tao et al. [80]	2010	China	Asian	0.41	Yes	0.95	0.19	608	0.96	0.18	939	0.97	0.18	272	6
Kappelle et al. [81]	2013	The Netherlands	Caucasian	0.42	Yes	1.28	0.37	2301	1.35	0.40	3233	1.41	0.42	1246	6
Li et al. [82]	2014	China	Asian	0.33	Yes	0.99	0.23	82	1.10	0.32	73	1.10	0.27	21	6
Galati et al. [83]	2014	Italia	Caucasian	0.42	Yes	1.52	0.45	73	1.45	0.30	106	1.61	0.42	39	7
El-Aziz et al. [50]	2014	Egypt	Caucasian	0.49	Yes	0.81	0.11	68	1.14	0.21	117	1.53	0.19	62	6
Zhai et al. [84]	2015	China	Asian	0.48	Yes	0.96	0.28	12	1.10	0.25	34	1.12	0.31	14	6
Jeenduang et al. [85]	2015	Thailand	Asian	0.37	Yes	1.34	0.32	152	1.35	0.35	169	1.39	0.31	57	6

USA: The United States, UK: United Kingdom, HWE: Hardy-Weinberg equilibrium, SD: standard deviation, HDL-C: High density lipoprotein cholesterol, MAF: minor allele frequencies.

**Table 3:** Metal-analysis of *CETP TaqIB* polymorphism and risk of atherosclerosis in each subgroup.

Position	Size (Case/Control)	Allele Model		Additive Model		Recessive Model		Dominant Model	
		OR (95% CI)	*p* Value	OR (95% CI)	*p* Value	OR (95% CI)	*p* Value	OR (95% CI)	*p* Value
**Overall analysis**	20,866/21,298	1.15 (1.09–1.21)	*p* < 0.001	1.26 (1.19–1.34)	*p* < 0.001	1.13 (1.08–1.18)	*p* < 0.001	1.20 (1.14–1.27)	*p* < 0.001
*Subgroup analysis based on ethnicity*									
Asian	5178/5084	1.24 (1.15–1.35)	*p* < 0.001	1.52 (1.35–1.72)	*p* < 0.001	1.41 (1.29–1.53)	*p* < 0.001	1.28 (1.15–1.42)	*p* < 0.001
Caucasian	15,688/16,214	1.09 (1.04–1.16)	0.001	1.19 (1.11–1.27)	*p* < 0.001	1.05 (1.00–1.11)	0.041	1.18 (1.11–1.25)	*p* < 0.001
*Subgroup analysis based on type of diseases*									
MI	9067/9393	1.10 (1.03–1.19)	0.009	1.18 (1.08–1.29)	*p* < 0.001	1.05 (0.99–1.12)	0.104	1.17 (1.08–1.26)	*p* < 0.001
IS	531/556	1.39 (1.17–1.66)	*p* < 0.001	1.92 (1.33–2.77)	0.001	1.40 (1.09–1.79)	*p* < 0.001	1.76 (1.25–2.47)	0.001
CAD	11,268/11,349	1.15 (1.08–1.24)	*p* < 0.001	1.31 (1.21–1.43)	*p* < 0.001	1.19 (1.12–1.27)	*p* < 0.001	1.21 (1.13–1.31)	*p* < 0.001
*Subgroup analysis based on source of controls*									
PB	14,735/11,618	1.11 (1.05–1.17)	*p* < 0.001	1.21 (1.13–1.29)	*p* < 0.001	1.09 (1.04–1.15)	0.001	1.17 (1.10–1.25)	*p* < 0.001
HB	6131/5180	1.20 (1.10–1.31)	*p* < 0.001	1.42 (1.26–1.59)	*p* < 0.001	1.24 (1.14–1.35)	*p* < 0.001	1.28 (1.16–1.42)	*p* < 0.001
*Subgroup analysis based on study type*									
CCS	15,155/15,187	1.14 (1.10–1.18)	*p* < 0.001	1.30 (1.21–1.39)	*p* < 0.001	1.16 (1.11–1.22)	*p* < 0.001	1.22 (1.15–1.30)	*p* < 0.001
CS	5711/6111	1.07 (1.01–1.13)	0.023	1.16 (1.03–1.30)	0.012	1.05 (0.97–1.14)	0.277	1.15 (1.04–1.28)	0.007
**Sensitivity analysis**									
BHWE	14,461/16,034	1.16 (1.09–1.23)	*p* < 0.001	1.33 (1.23–1.42)	*p* < 0.001	1.18 (1.12–1.24)	*p* < 0.001	1.24 (1.16–1.32)	*p* < 0.001
BS	18,902/19,454	1.12 (1.08–1.15)	*p* < 0.001	1.25 (1.18–1.33)	*p* < 0.001	1.13 (1.08–1.18)	*p* < 0.001	1.20 (1.14–1.27)	*p* < 0.001

CAD: coronary artery disease, MI: myocardial infraction, IS: ischemic stroke, HB: hospital-based, PB: population-based, HWE: Hardy-Weinberg equilibrium, CS: cohort study, CCS: case control study, BHWE: based on Hardy-Weinberg equilibrium (Studies without Hardy-Weinberg equilibrium were excluded), BS: based on sample size (Studies with sample size < 400 were excluded).

## Supplementary Materials

The following are available online at www.mdpi.com/1660-4601/13/9/882/s1, Figure S1: Meta-analysis of the composite ischemic CVD and the *CETP TaqIB* polymorphism (additive genetic model: *B1B1* vs. *B2B2*). Figure S2: Meta-analysis of the composite ischemic CVD and the *CETP TaqIB* polymorphism (dominate genetic model: *B1B1*+*B1B2* vs. *B2B2*). Figure S3: Meta-analysis of the composite ischemic CVD and the *CETP TaqIB* polymorphism (recessive genetic model: B1B1 vs. *B1B2* + *B2B2*). Figure S4: Association between the *CETP TaqIB* polymorphism and HDL-C concentrations (*B1B1* vs. *B1B2*). Figure S5: Association between the *CETP TaqIB* polymorphism and HDL-C concentrations (*B1B2* vs. *B1B2*). Figure S6: Sensitivity analysis based on sample size for the associations between the *CETP TaqIB* polymorphism and HDL-C concentrations (*B1B1* vs. *B2B2*). Figure S7: Sensitivity analysis based on Hardy–Weinberg equilibrium for the associations between the *CETP TaqIB* polymorphism and HDL-C concentrations (*B1B1* vs. *B2B2*). Figure S8: Sensitivity analysis based on sample size for the associations between the *CETP TaqIB* polymorphism and HDL-C concentrations (*B1B2* vs. *B2B2*). Figure S9: Sensitivity analysis based on Hardy–Weinberg equilibrium for the associations between the *CETP TaqIB* polymorphism and HDL-C concentrations (*B1B2* vs. *B2B2*). Figure S10: Sensitivity analysis based on sample size for the associations between the *CETP TaqIB* polymorphism and HDL-C concentrations (*B1B1* vs. *B1B2*). Figure S11: Sensitivity analysis based on Hardy–Weinberg equilibrium for the associations between the *CETP TaqIB* polymorphism and HDL-C concentrations (*B1B1* vs. *B1B2*). Figure S12: Analysis of heterogeneity for Asian studies by Galbraith plot (*B1B1* vs. *B2B2*). Figure S13: Analysis of heterogeneity for Caucasian studies by Galbraith plot (*B1B1* vs. *B2B2*). Figure S14: Association between the *CETP TaqIB* polymorphism and HDL-C concentrations after exclusion of these outlier studies (*B1B1* vs. *B2B2*). Figure S15: Association between the *CETP TaqIB* polymorphism and HDL-C concentrations after exclusion of these outlier studies (*B1B1* vs. *B1B2*). Figure S16: Association between the *CETP TaqIB* polymorphism and HDL-C concentrations after exclusion of these outlier studies (*B1B2* vs. *B2B2*). Checklist S1: PRISMA 2009 checklist. Table S1: MOOSE checklist.
