# Supplementary material for: Associations of Cholesteryl Ester Transfer Protein TaqIB Polymorphism with the Composite Ischemic Cardiovascular Disease Risk and HDL-C Concentrations: A Meta-Analysis
Source: Int J Environ Res Public Health. 2016 Sep 5;13(9):882. doi: 10.3390/ijerph13090882 (PMC5036715; doi:10.3390/ijerph13090882)
Supplement: Supplementary file 1 [file ijerph-13-00882-s001.pdf]

Supplementary Materials: Associations of Cholesteryl Ester Transfer Protein TaqIB Polymorphism with the Composite Ischemic Cardiovascular Disease Risk and HDL-C Concentrations: A Meta-Analysis

Shu-xia Guo, Ming-hong Yao, Yu-song Ding, Jing-yu Zhang, Yi-zhong Yan, Jia-ming Liu, Mei Zhang, Dong-sheng Rui, Qiang Niu, Jia He, Heng Guo and Ru-lin Ma

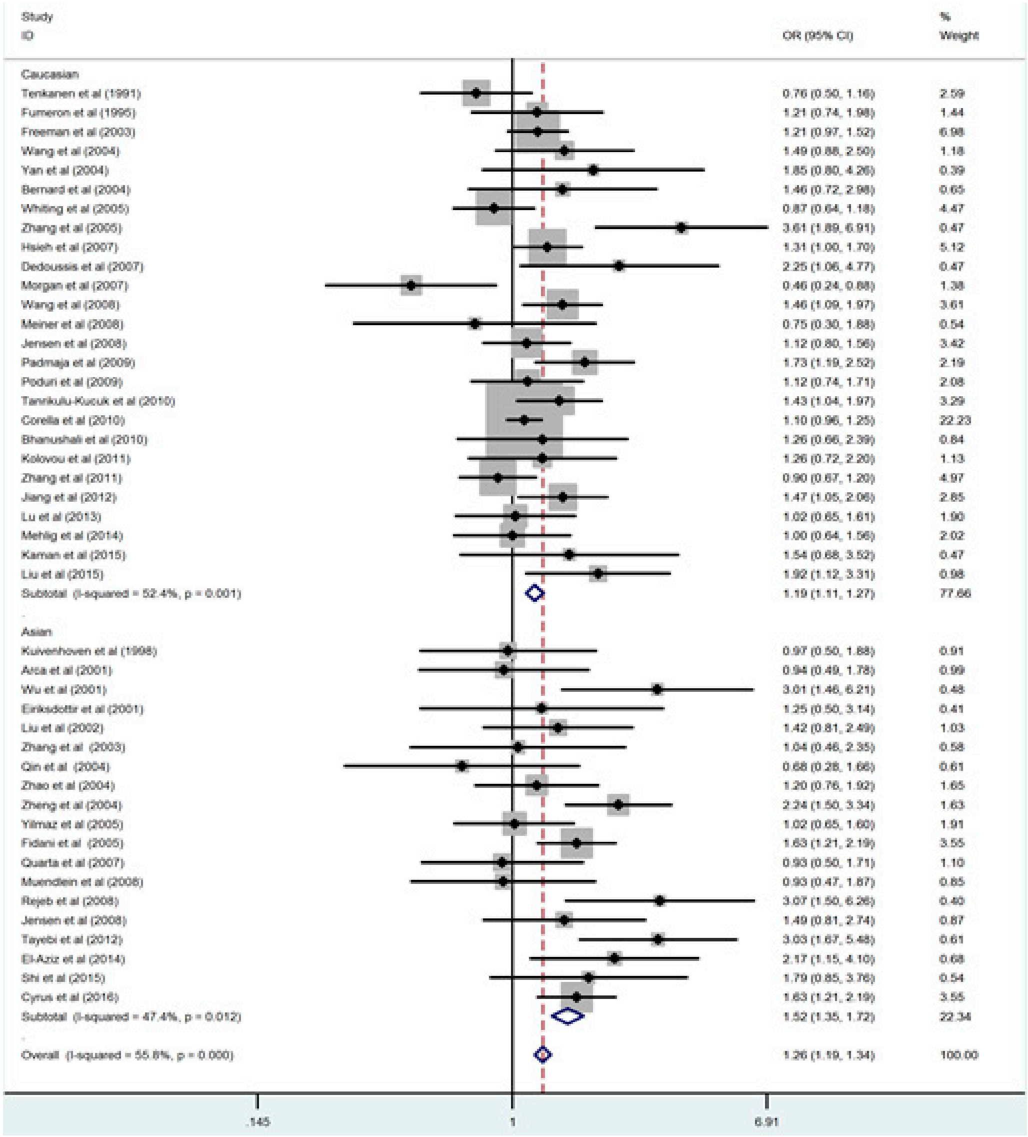

Figure S1. Meta-analysis of the composite ischemic CVD and the *CETP TaqIB* polymorphism (additive genetic model: *B1B1* vs. *B2B2*).

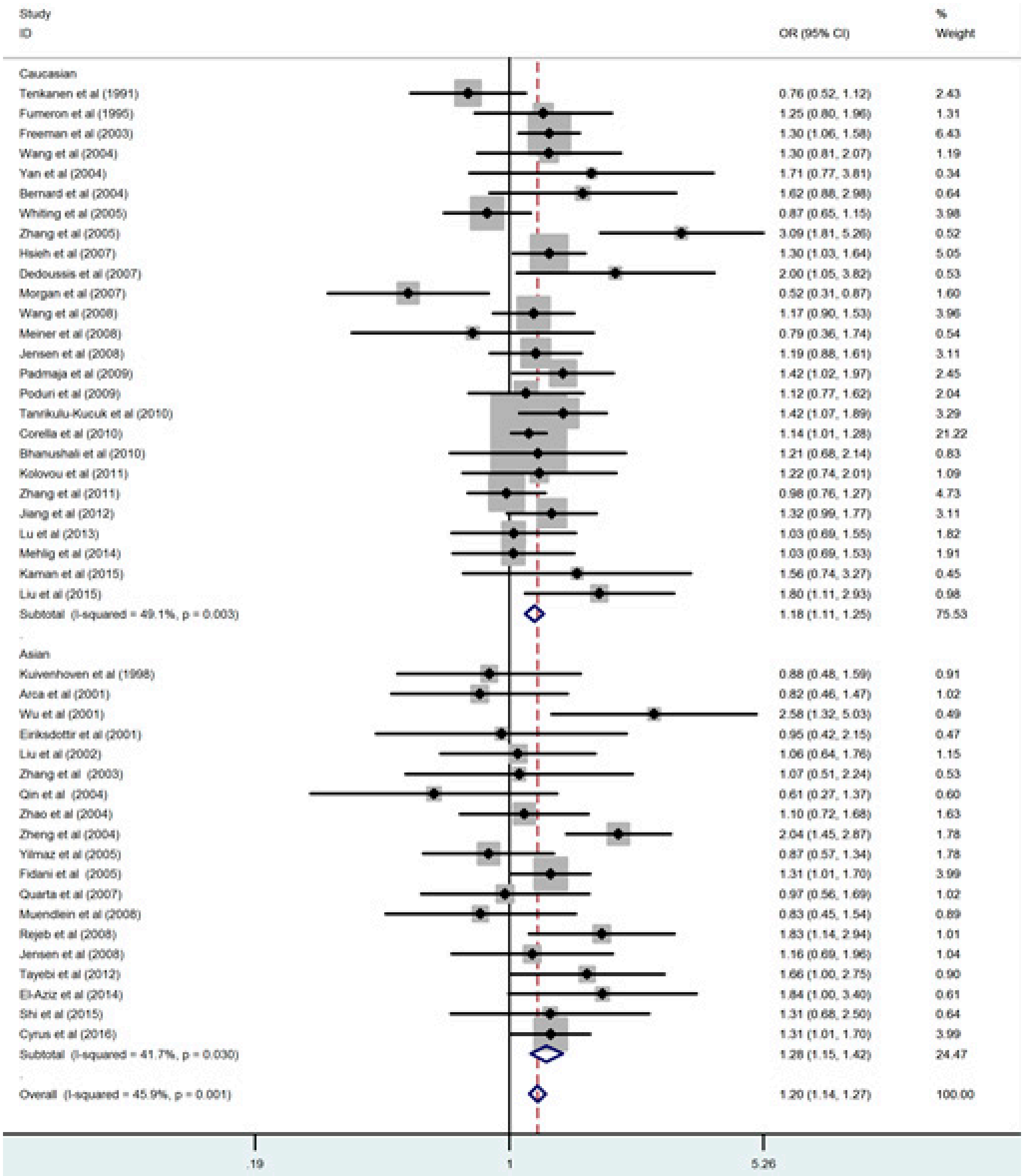

Figure S2. Meta-analysis of the composite ischemic CVD and the *CETP TaqIB* polymorphism (dominate genetic model: *B1B1* + *B1B2* vs. *B2B2*).

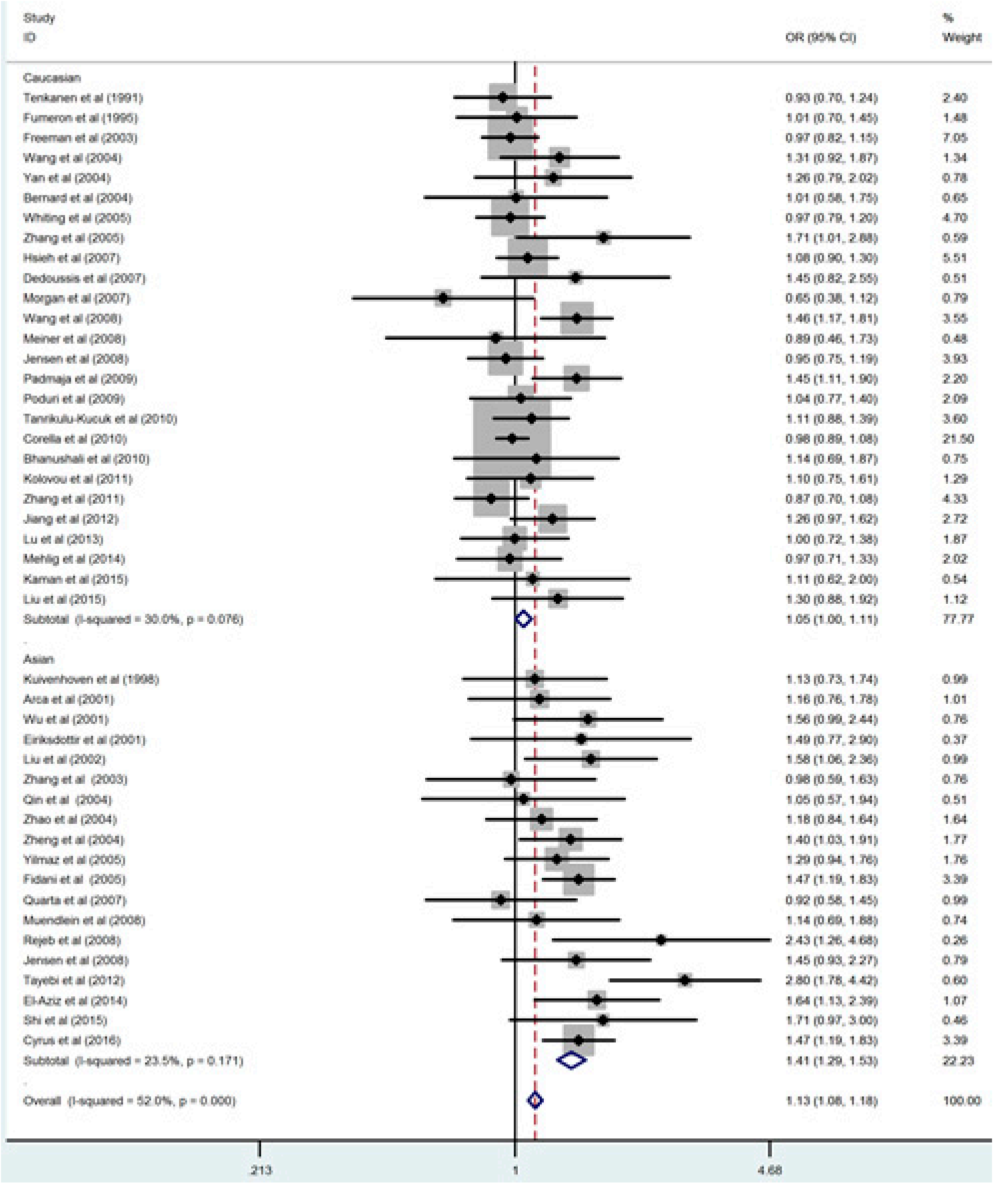

Figure S3. Meta-analysis of the composite ischemic CVD and the *CETP TaqIB* polymorphism (recessive genetic model: *B1B1* vs. *B1B2* + *B2B2*).

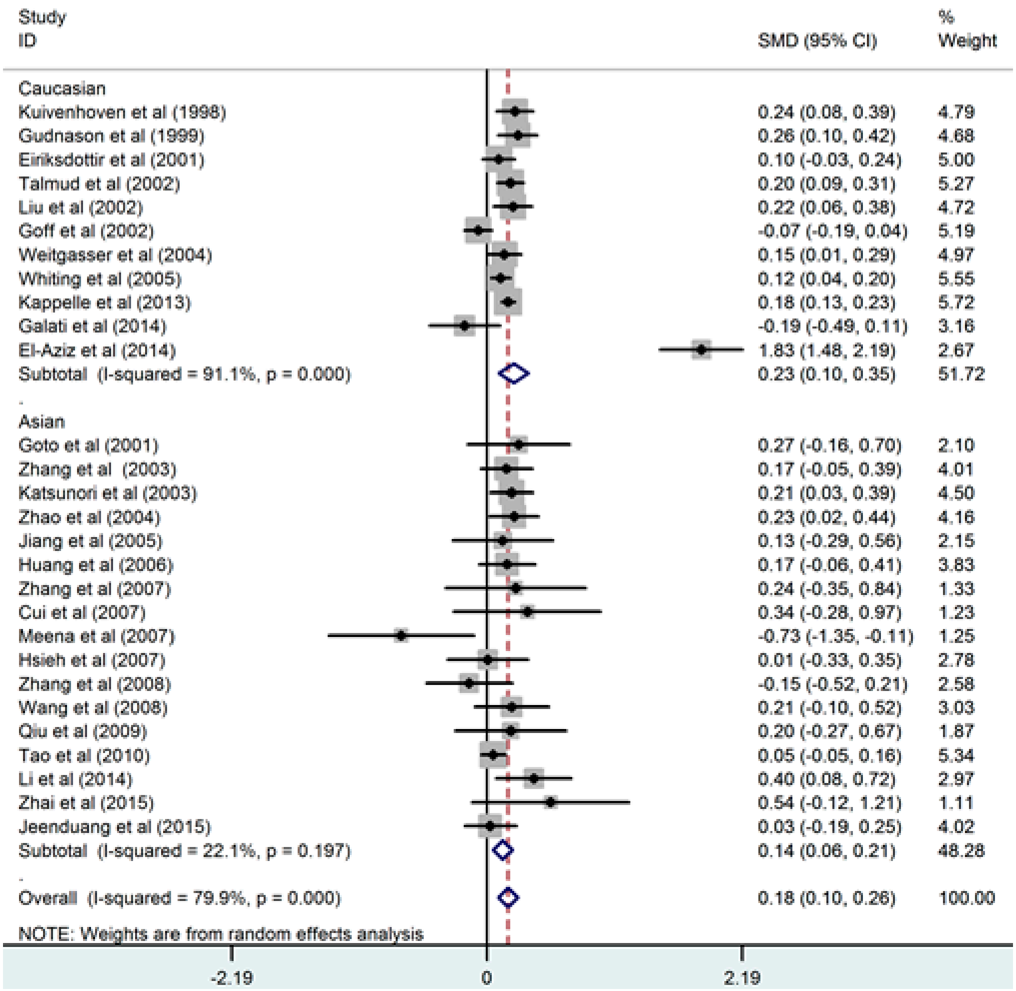

Figure S4. Association between the *CETP TaqIB* polymorphism and HDL-C concentrations (*B1B1* vs. *B1B2*).

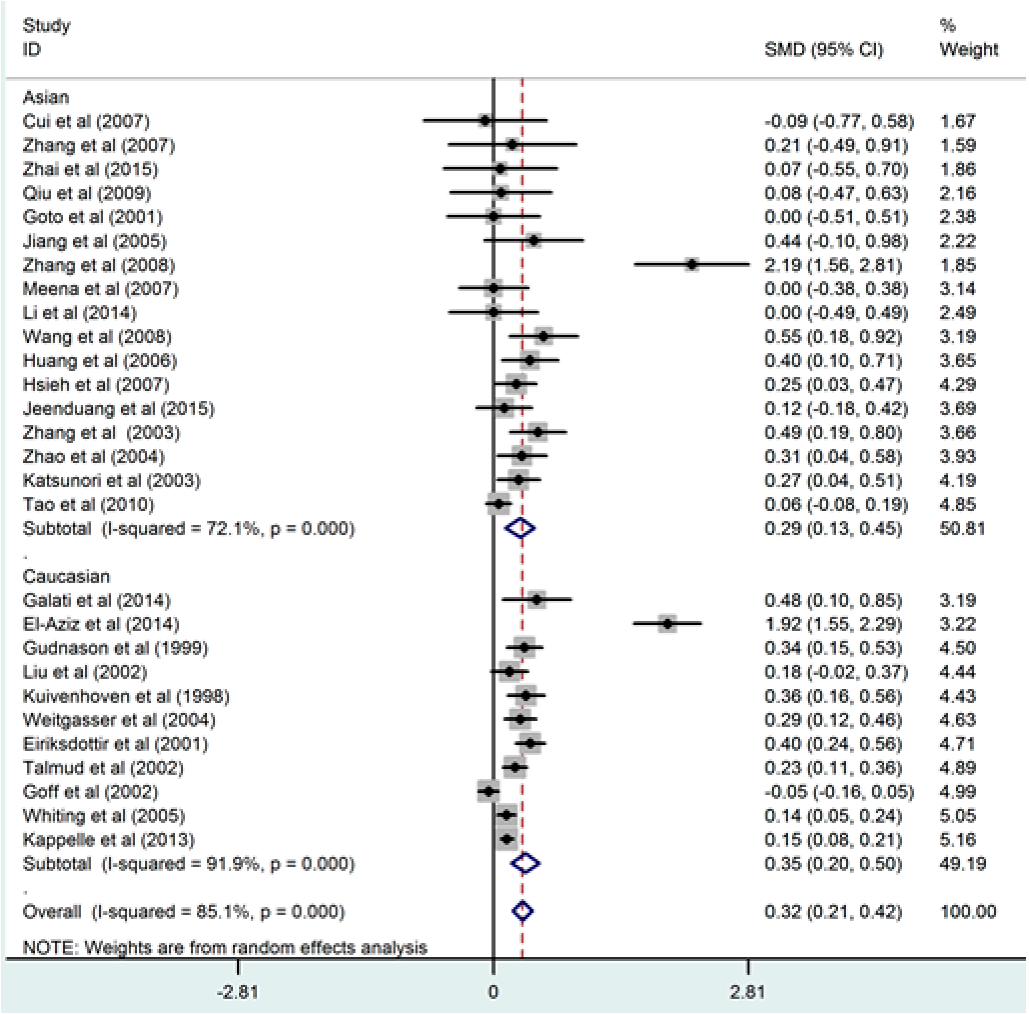

Figure S5. Association between the *CETP TaqIB* polymorphism and HDL-C concentrations (*B1B2* vs. *B1B2*).

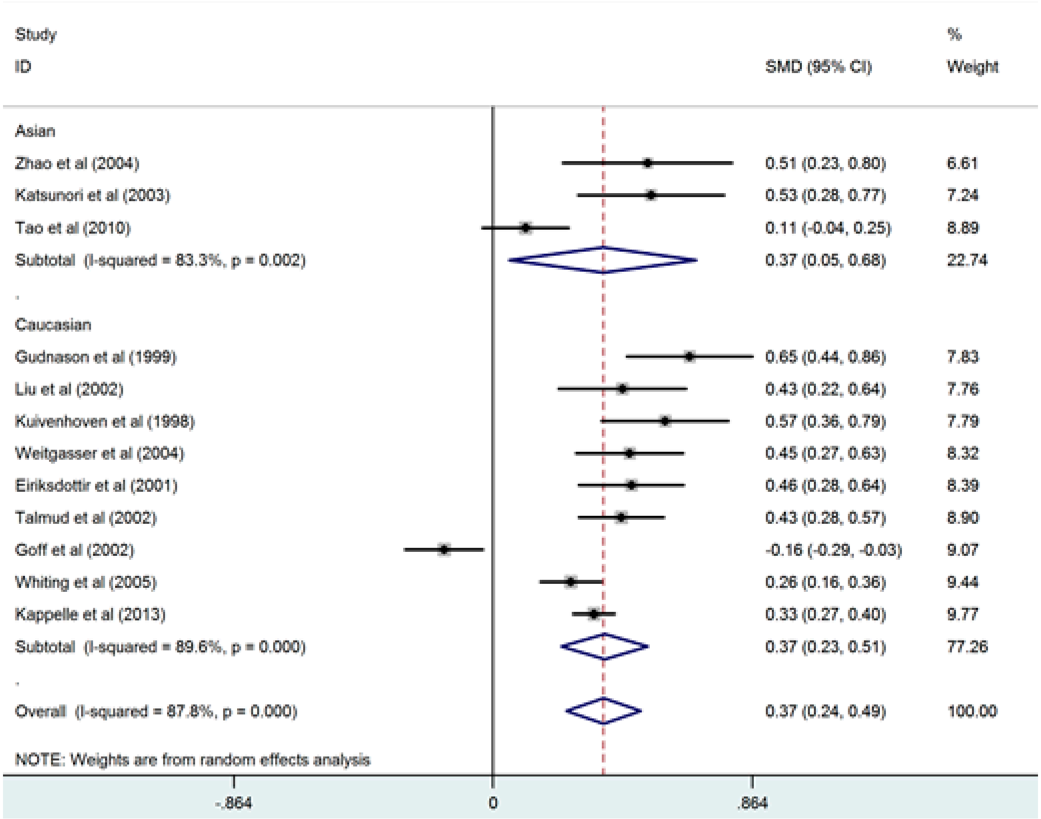

Figure S6. Sensitivity analysis based on sample size for the associations between the *CETP TaqIB* polymorphism and HDL-C concentrations (*B1B1* vs. *B2B2*).

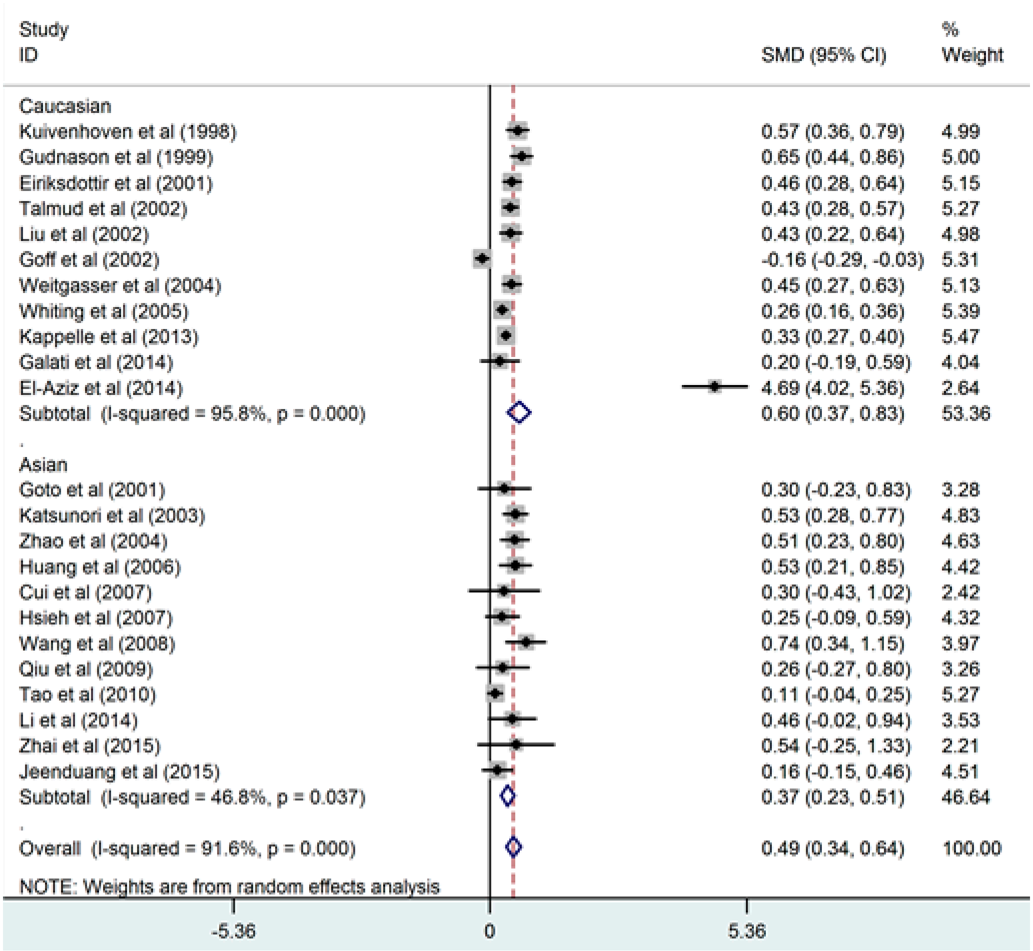

Figure S7. Sensitivity analysis based on Hardy–Weinberg equilibrium for the associations between the *CETP TaqIB* polymorphism and HDL-C concentrations (*B1B1* vs. *B2B2*).

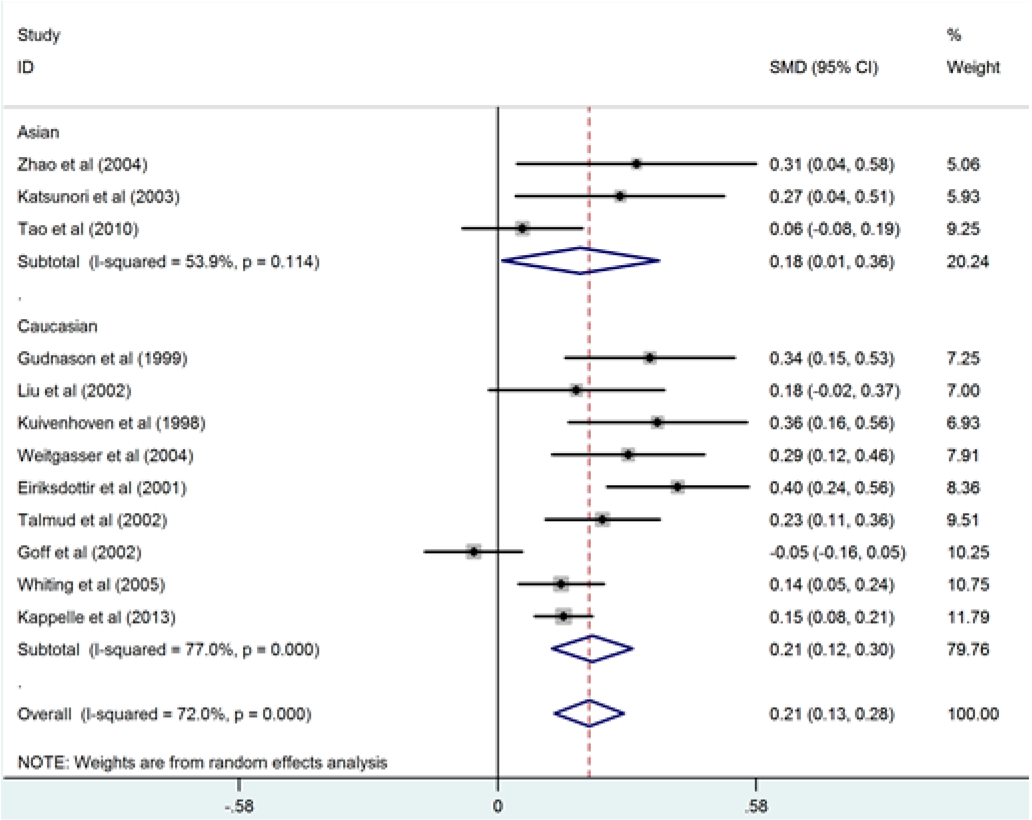

Figure S8. Sensitivity analysis based on sample size for the associations between the *CETP TaqIB* polymorphism and HDL-C concentrations (*B1B2* vs. *B2B2*).

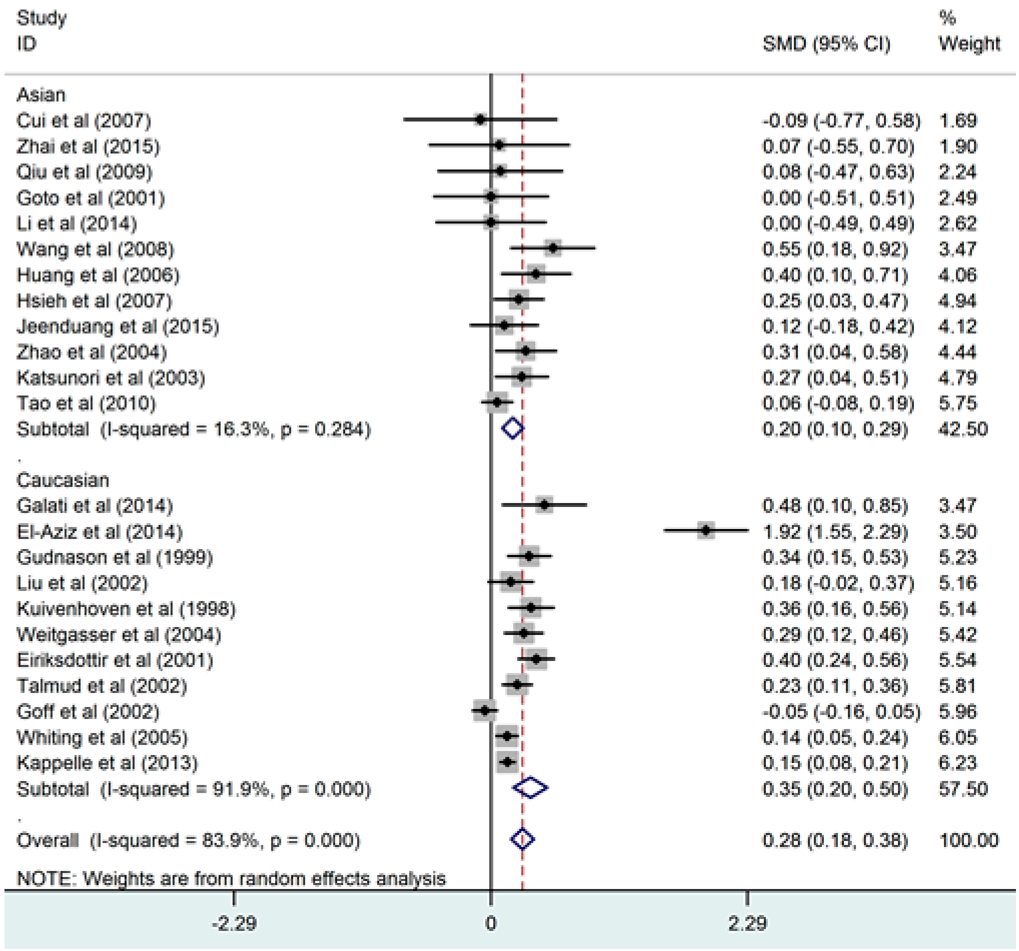

Figure S9. Sensitivity analysis based on Hardy–Weinberg equilibrium for the associations between the *CETP TaqIB* polymorphism and HDL-C concentrations (*B1B2* vs. *B2B2*).

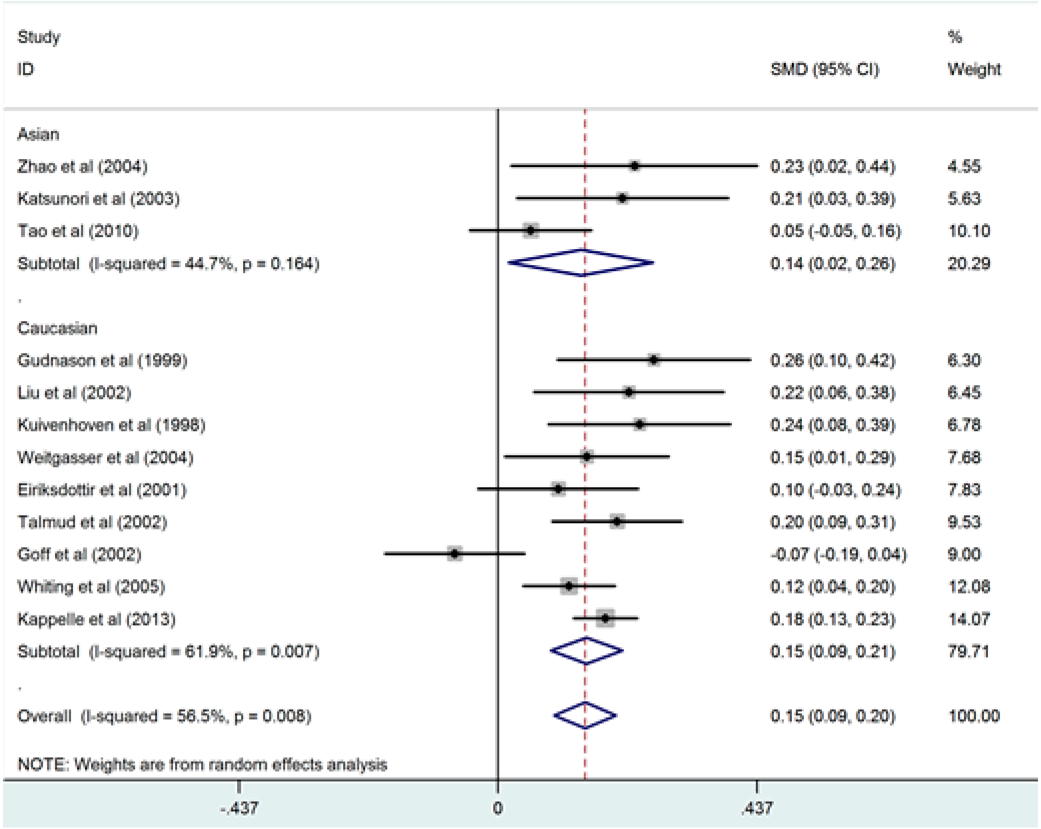

Figure S10. Sensitivity analysis based on sample size for the associations between the *CETP TaqIB* polymorphism and HDL-C concentrations (*B1B1* vs. *B1B2*).

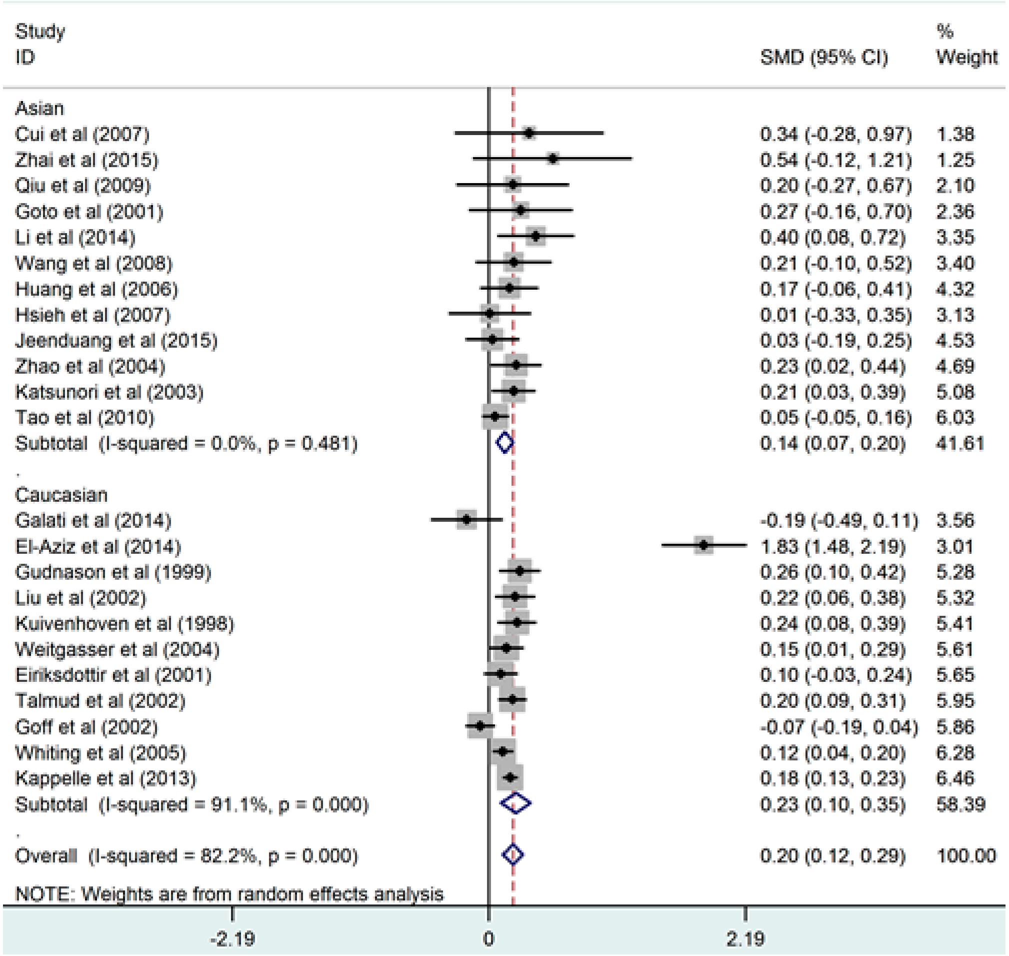

Figure S11. Sensitivity analysis based on Hardy–Weinberg equilibrium for the associations between the *CETP TaqIB* polymorphism and HDL-C concentrations (*B1B1* vs. *B1B2*).

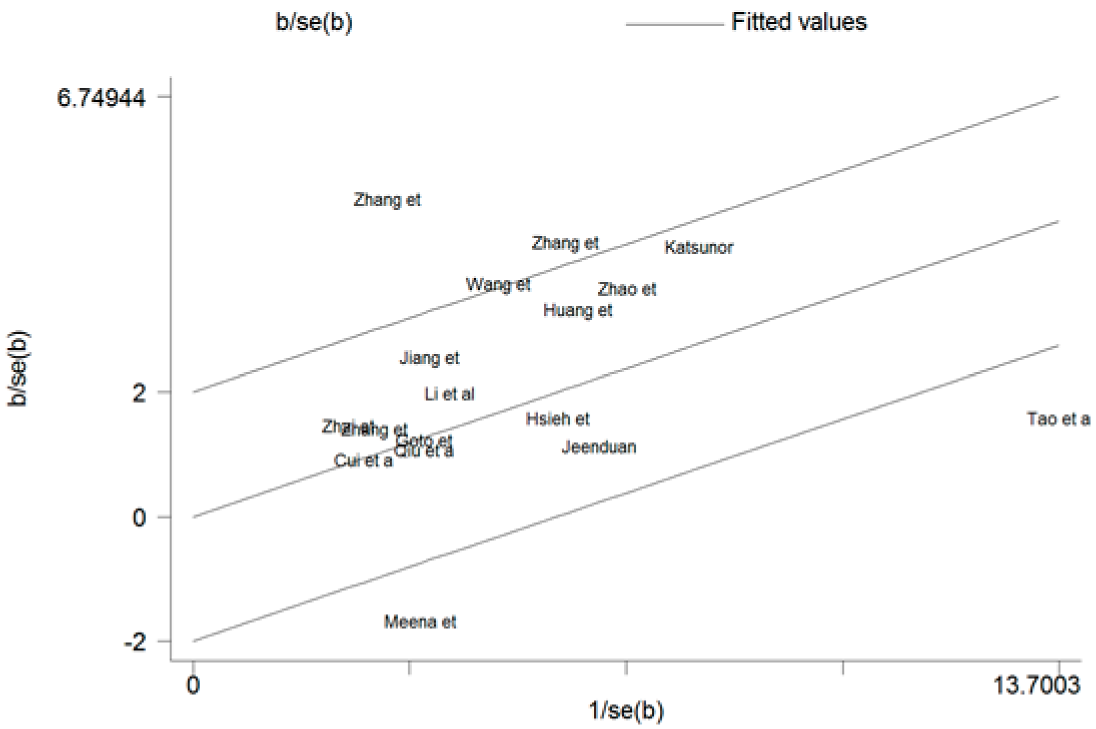

Figure S12. Analysis of heterogeneity for Asian studies by Galbraith plot (*B1B1* vs. *B2B2*).

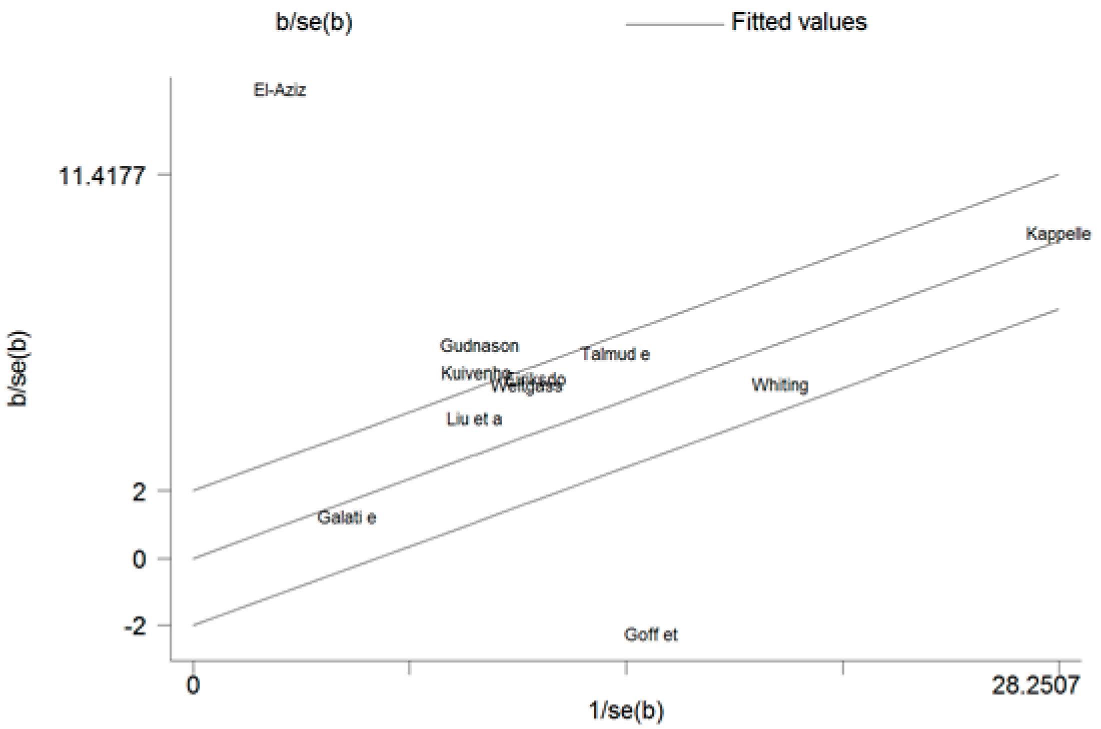

Figure S13. Analysis of heterogeneity for Caucasian studies by Galbraith plot (*B1B1* vs. *B2B2*).

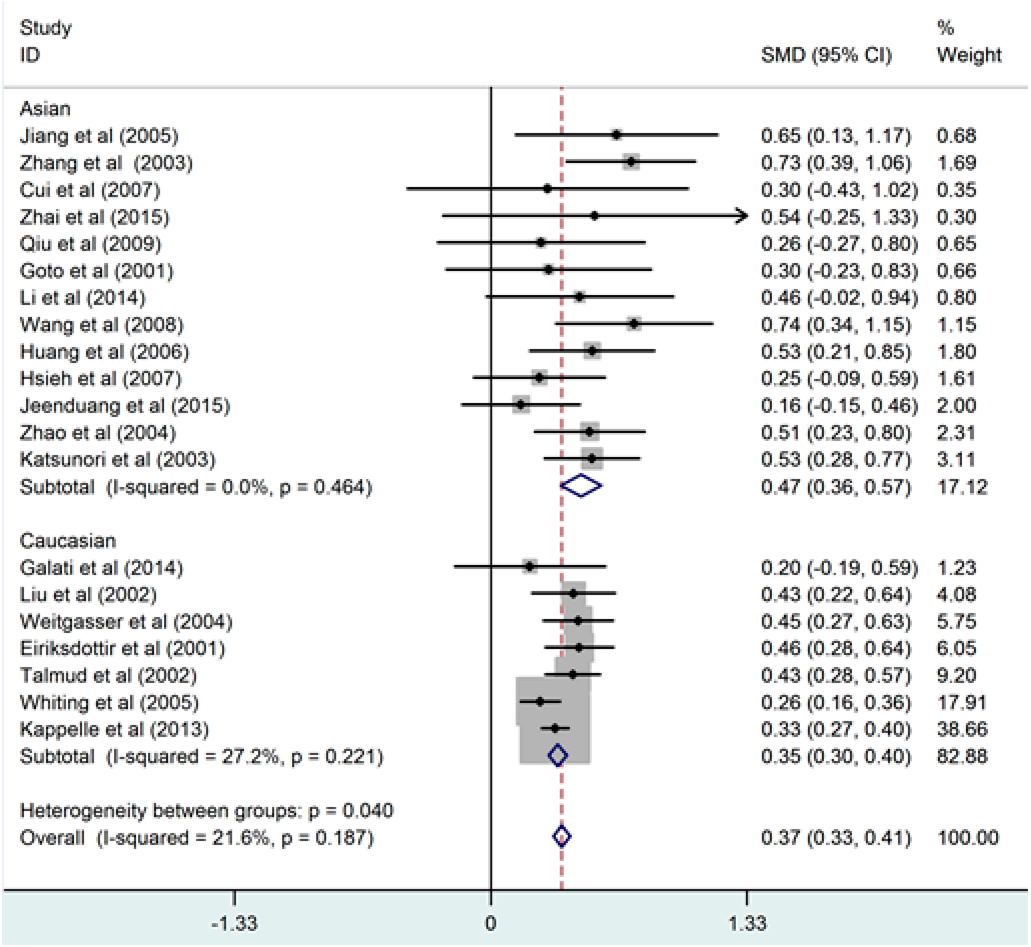

Figure S14. Association between the *CETP TaqIB* polymorphism and HDL-C concentrations after exclusion of these outlier studies (*B1B1* vs. *B2B2*).

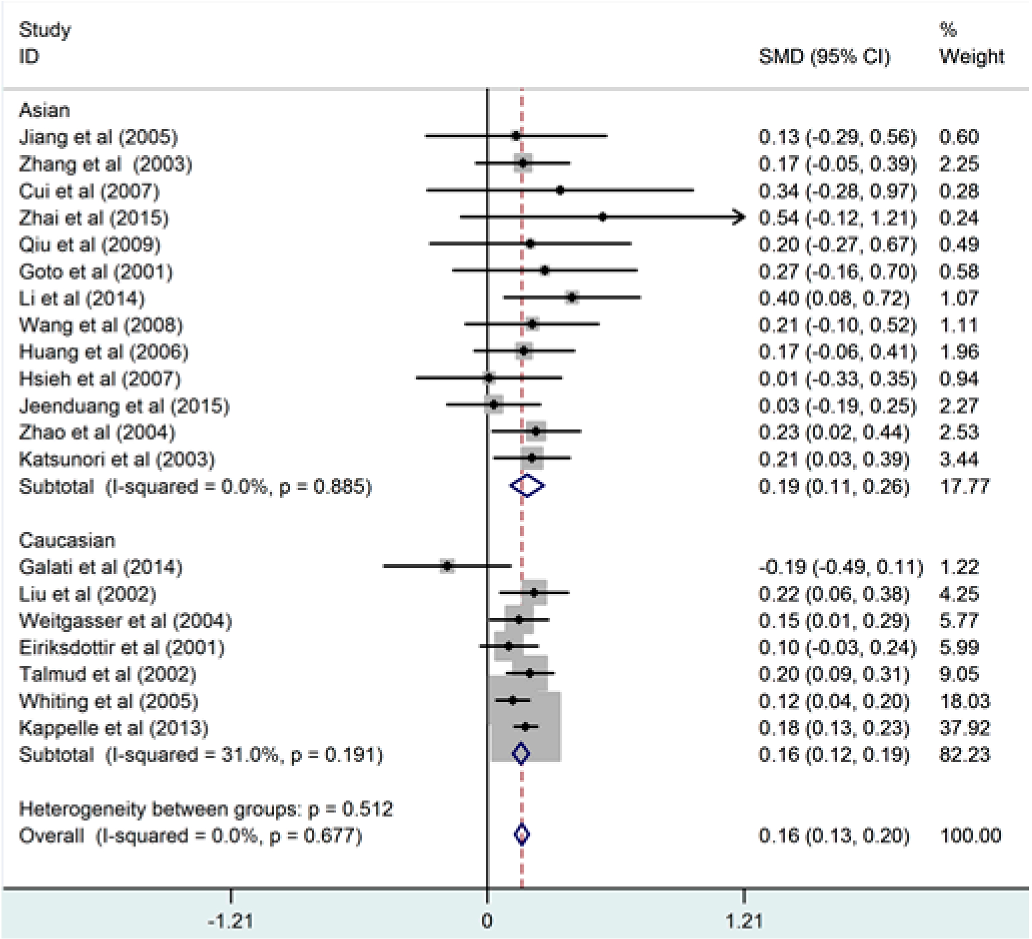

Figure S15. Association between the *CETP TaqIB* polymorphism and HDL-C concentrations after exclusion of these outlier studies (*B1B1* vs. *B1B2*).

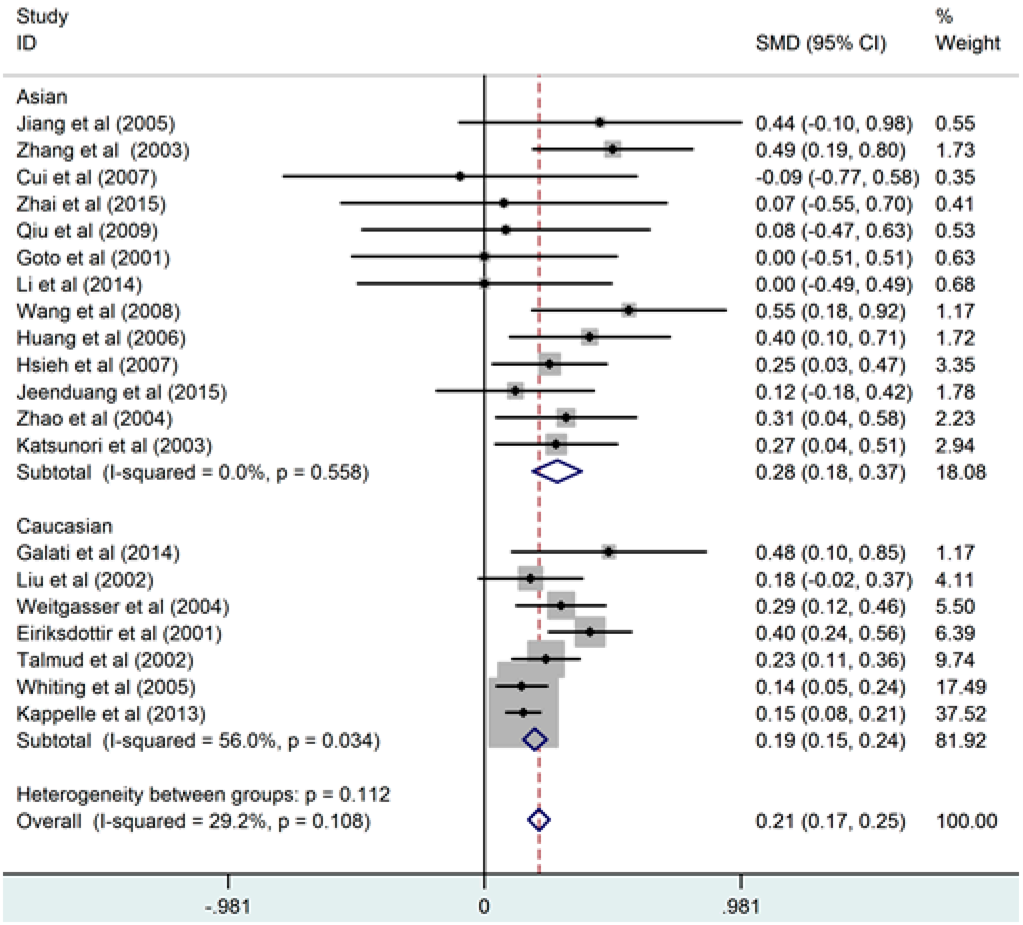

Figure S16. Association between the *CETP TaqIB* polymorphism and HDL-C concentrations after exclusion of these outlier studies (*B1B2* vs. *B2B2*).

Table S1. MOOSE Checklist.

	Criteria	Brief Description of How the Criteria Were Handled in the Meta-Analysis
**Reporting of background should include**		
√	Problem definition	*CETP TaqIB* polymorphism is closely associated with HDL-C level and various diseases including CAD, IS and MI. However, the associations between *CETP TaqIB* polymorphism and serum HDL-C level and susceptibility to AS were inconsistent in previous studies.
√	Hypothesis statement	It is likely that *CETP TaqIB* polymorphism may influence the serum HDL-C level and susceptibility of AS.
√	Description of study outcomes	Atherosclerosis
√	Type of exposure or intervention used	For the association between *CETP TaqIB* polymorphism and AS, *B1B1* vs. *B2B2*, *B1B1* + *B1B2* vs. *B2B2* and *B1B1* vs. *B1B2* + *B2B2* genotypes or *B1* vs. *B2* allele; For the association between *CETP TaqIB* polymorphism and HDL-C, *B1B1* vs. *B2B2*, *B1B1* vs. *B1B2*, and *B1B2* vs. *B2B2*.
√	Type of study designs used	Published case-control, nested case-control or cohort designs studies.
√	Study population	No restriction.
**Reporting of search strategy should include**		
√	Qualifications of searchers	Investigators include experts in atherosclerotic diseases and qualified graduate students. All of the investigators have received training in literature research, statistics and evidence-based medicine.
√	Search strategy, including time period included in the synthesis and keywords	We selected possibly relevant articles in the Cochrane Library, Embase, PubMed, Web of Science, Springer, China Science and Technology Journal Database (CSTJ), China National Knowledge Infrastructure (CNKI), Google Scholar and Baidu Library (last search conducted in January 2016) with search strategy: ("Cholesterol ester transfer protein" OR "CETP") and ("variation" OR "variant" OR "mutation" OR "polymorphism" OR "genotype") and ("CAD" OR "coronary artery disease" OR "coronary heart disease" OR "CHD" OR "myocardial infarction" OR "MI" OR "ischemic cardiovascular disease" OR "IS") and ("high-density lipoprotein cholesterol" OR "HDL-C" OR "blood lipid" OR "serum lipid").
√	Databases and registries searched	The Cochrane Library, Embase, PubMed, Web of Science, Springer, China Science and Technology Journal Database (CSTJ), China National Knowledge Infrastructure (CNKI), Google Scholar and Baidu Library
√	Search software used, name and version, including special features	We did not employ any search software.
	Use of hand searching	Other relevant studies were identified by hand-searching the references of included articles identified by electronic search.
√	List of citations located and those excluded, including justifications	Literature search and selection process are outlined in the flow diagram. The reasons for exclusion were listed in the flow diagram and explained in result section.
	Method of addressing articles published in languages other than English	The search was limited to English and Chinese language papers.

√	Method of handling abstracts and unpublished studies	We first examined if overlap existed and excluded overlapped studies. We only included published case-control, nested case-control or cohort designs studies.
√	Description of any contact with authors	If necessary data were not reported in the primary manuscripts, we contacted the corresponding authors by email to request the missing data.
**Reporting of methods should include**		
√	Description of relevance or appropriateness of studies assembled for assessing the hypothesis to be tested	Eligibility criteria: The eligibility criteria for including articles in the present meta-analysis were as following major criteria: (1) the publication evaluating the associations of the CETP TaqIB polymorphism with AS or HDL-C level; (2) all atherosclerosis cases were diagnosed were made according to the internationally recognized diagnostic criterion as follows: criteria of World Health Organization (WHO), criteria of American College of Cardiology/American Heart Association (ACC/AHA), criteria of European Society of Cardiology (ESC), or angiographic coronary stenosis (generally defined as at least 50% stenosis of one major coronary artery); (3) published in either Chinese or English; (4) for CAD association, sufficient published data for calculating odds ratios (ORs) with their 95% confidence intervals (CIs); for HDL-C level association, the number of population, the mean of HDL-C level and the standard deviations (SD) by genotypes should be available.<br>Exclusion criteria*:* The exclusion criteria were as follows: (1) Duplicate publications; (2) incomplete information; (3) insufficient or insignificant statistical data; (4) review articles.
√	Rationale for the selection and coding of data	For the association between *CETP TaqIB* polymorphism and AS, We used the crude ORs and 95% CIs for meta-analysis. If the studies did not provide crude ORs and 95% CIs, we calculated the ORs and 95% CIs by the total numbers of cases and controls, and frequencies of *CETP TaqIB* polymorphism in cases and controls.<br>For the association between *CETP TaqIB* polymorphism and HDL-C, a pooled standardized mean difference (SMD) and its 95% CIs were used for the meta-analysis.
√	Assessment of confounding	NOS rating system was used to assess the confounder. Subgroup analyses were performed and sensitivity analyses were also performed.
√	Assessment of study quality, including blinding of quality assessors; stratification or regression on possible predictors of study results	We assessed the methodological qualities of included studies by the description of study population, the set of controls and cases and related statistical methods. We carried out sensitivity analysis.
√	Assessment of heterogeneity	Heterogeneity was assessed by the Q-test and I^2 statistic, $p < 0.10$ and $I^2 > 50\%$ indicated evidence of heterogeneity.
√	Description of statistical methods in sufficient detail to be replicated	Methods of heterogeneity test, quantitative synthesis, assessments of publication bias, sensitivity analyses were reported in detail in the methods section.
√	Provision of appropriate tables and graphics	We provided flow chart to explain literature searching and selection (Figure 1); forest plots for the total analysis, (Figures 2 and 3, Figures S1–S5); study characteristics and allele/genotype frequencies (Tables 1 and 2).
**Reporting of results should include**		
	Graph summarizing individual study estimates and overall estimate	Graph summarizing individual study estimates and overall estimate are presenting in Figures 2 and 3, Figures S1–S5.

√	Table giving descriptive information for each study included	Descriptive information for each study included was provided in Tables 1 and 2.
√	Results of sensitivity testing	The results of sensitivity analysis were described in results section. Table 3 provided detailed results for the sensitivity analyses.
√	Indication of statistical uncertainty of findings	The results of heterogeneity test, pooled ORs, 95% confidence intervals and p value for Z test were presented with all pooled analyses.
**Reporting of discussion should include**		
√	Quantitative assessment of bias	We evaluated the publication bias by funnel plots, egger's test.
√	Justification for exclusion	Based on our preliminary search criteria, a total of 478 publications were eligible. Among these studies, 279 records were excluded (reviews, no out of interest, meta-analysis, duplicate publication and records not published in Chinese and English) and 134 articles without original data were excluded.
√	Assessment of quality of included studies	We discussed the results of sensitivity analyses and described the limitations of included studies.
**Reporting of conclusions should include**		
√	Consideration of alternative explanations for observed results	We discussed that potential unmeasured confounders and explained the limitations of this meta-analysis. We reminded readers that caution should be made when interpreting this meta-analysis.
√	Generalization of the conclusions	Our meta-analysis suggested that the *CETP TaqIB* polymorphism were associated with serum HDL-C level and the susceptibility to AS.
√	Guidelines for future research	Larger sample-size studies with homogeneous AS patients and well-matched controls are required.
√	Disclosure of funding source	This study was supported by grants from National Science and Technology Support Projects for the "Eleventh Five-Years Plan" of China (No. 2009BAI82B04), National Natural Science Foundation of China (No. 81560551) and Special Fund for Investigation of chronic heart and lung disease in Tibet and Xinjiang of China (N0. 201402002).

PRISMA 2009 Checklist

Section/topic	#	Checklist Item	Reported on Page #
**TITLE**			
**Title**	1	Associations of the Cholesteryl Ester Transfer Protein TaqIB polymorphism with atherosclerosis risk and HDL-C level: A meta-analysis	1
**ABSTRACT**			
**Structured summary**	2	**Background:** Previous studies have evaluated the associations of the Cholesterol ester transfer protein (CETP) TaqIB polymorphism (rs708272) with the risk of developing atherosclerosis (AS) and the level of high-density lipoprotein cholesterol (HDL-C), but results remain controversial. The objective of this study is to investigate whether there was relationship of the *CETP TaqIB* polymorphism with the level of AS and HDL-C using a meta-analysis. **Methods:** We conducted a meta-analysis of available studies to clarify the associations of the *CETP TaqIB* polymorphism with HDL-C level and AS risk. All statistical analyses were done with Stata 12.0. **Results:** Through retrieving the Cochrane Library, Embase, PubMed, Web of Science, Springer, China Science and Technology Journal Database, China National Knowledge Infrastructure, Google Scholar and Baidu Library, we identified a total of 45 studies from 44 studies with 20,866 cases and 21,298 controls were combined showing significant association between the *CETP TaqIB* variant and AS risk, the carriers of allele TaqIB-B1 were found to have a higher risk of AS than the non-carriers: OR = 1.15, 95% CI = 1.09–1.21, $p < 0.001$; meanwhile, 28 studies with 23,959 subjects were included in the association between the *CETP TaqIB* polymorphism and the level of HDL-C, It was suggested that the carriers of B1B1 genotype had lower level of HDL-C than those of B2B2 genotype: SMD = 0.50, 95% CI = 0.36–0.65, $p < 0.001$. **Conclusions:** The synthesis of available evidence demonstrates that the *CETP TaqIB* polymorphism is a protective role for AS risk both in Asians and Caucasians and also associated with a higher HDL-C level in Asians and Caucasians.	2
**INTRODUCTION**			
**Rationale**	3	Coronary artery disease (CAD) and Myocardial infarction (MI) have become the serious public health problems in the world because of its high morbidity and mortality [1,2]. However, its exact mechanisms are still unclear. For a long time, atherosclerosis has attracted more attention because of it's the pathological foundation of CAD and MI. Abnormal cholesterol metabolism was considered to be main factor for atherosclerosis, and many epidemiological evidence has shown that low concentration of serum high-density lipoprotein cholesterol (HDL-C) was considered as an independent risk factors for atherosclerosis [3,4]. High-density lipoprotein (HDL) was demonstrated to play a pivotal role in mediating the transfer of cholesterol from extra hepatic tissues to the liver, and reducing the deposition of cholesterol in the artery wall [5]. Cholesterol ester transfer protein (CETP) gene located on chromosome 16q21, and encodes the key plasma protein that mediate the transfer of esterified cholesterol from HDL to apolipoprotein B-containing particles in exchange for triglycerides [6,7]. CETP gene mutation may affect the transcription and expression of the protein, thereby affecting serum HDL-C level [8]. *CETP TaqIB* (rs708272) polymorphism as the most common of CETP gene polymorphism loci has received a great deal of attention [9]. In recent years, numerous studies have showed relationship the CETP gene TaqIB polymorphism in the synthesis of HDL-C and AS risk, however, still remain inconsistent, possibly due to small sample sizes in the individual studies.	2
**Objectives**	4	In 2005, Boekholdt et al. performed a meta-analysis to evaluate the association the *CETP TaqIB* polymorphism in the synthesis of serum HDL-C and CAD risk, and demonstrated that the *CETP TaqIB* variant is associated with HDL-C level and CAD risk in Caucasians [10]. Li et al. also conducted a meta-analysis to evaluate the association of this variant with CAD in Chinese	3

		[11]. However, they were not observed the relationship between CETP TaqIB polymorphism and CAD. Cao et al. and Wang et al. performed meta-analysis to evaluate the association the CETP TaqIB variant and MI, their results shown that the CETP TaqIB-B2 allele is a protective factor to against the development of MI [12,13]. Considering the above four meta-analyses only focused on the association of CETP TaqIB polymorphism with the single atherosclerotic disease, we therefore performed this meta-analysis to clarify the role of the CETP gene TaqIB polymorphism in the synthesis of HDL-C and AS risk.	
**METHODS**			
**Protocol and registration**	5	**No protocol and registration.**	
**Eligibility criteria**	6	The eligibility criteria for including articles in the present meta-analysis were as following major criteria: (1) the publication evaluating the associations of the *CETP TaqIB* polymorphism with AS or HDL-C level; (2) all atherosclerosis cases were diagnosed were made according to the internationally recognized diagnostic criterion as follows: criteria of World Health Organization (WHO), criteria of American College of Cardiology/American Heart Association (ACC/AHA), criteria of European Society of Cardiology (ESC), or angiographic coronary stenosis (generally defined as at least 50% stenosis of one major coronary artery); (3) published in either Chinese or English; (4) for CAD association, sufficient published data for calculating odds ratios (ORs) with their 95% confidence intervals (CIs); for HDL-C level association, the number of population, the mean of HDL-C level and the standard deviations (SD) by genotypes should be available.<br>The exclusion criteria were as follows: (1) Duplicate publications; (2) incomplete information; (3) insufficient or insignificant statistical data; (4) review articles.	3-4
**Information sources**	7	Eligible literatures published before the end of January 2016 were identified by the search of the Cochrane Library, Embase, PubMed, Web of Science, Springer, China Science and Technology Journal Database (CSTJ), China National Knowledge Infrastructure (CNKI), Google Scholar and Baidu Library.	3
**Search**	8	Following Medical Subject Heading (MeSH) terms and/or text words were used for searching: (“Cholesterol ester transfer protein” OR “CETP”) and (“variation” OR “variant” OR “mutation” OR “polymorphism” OR “genotype”) and (“CAD” OR “coronary artery disease” OR “coronary heart disease” OR “CHD” OR “myocardial infarction” OR “MI” OR “ischemic cardiovascular disease” OR “IS”) and (“high-density lipoprotein cholesterol” OR “HDL-C” OR “blood lipid” OR “serum lipid”).	3
**Study selection**	9	The flow diagram of the study selection for this meta-analysis was shown in the Figure 1. 44 studies with 20866 cases and 21,298 controls were met the inclusion criteria and included to assess the association between the *CETP TaqIB* polymorphism and atherosclerosis [23–65]. Among these studies, there were 28 studies involving CAD [23–32,34-39,44,46,47,50,52–55,59–61,66] and 3 studies involving IS [63–65] and 12 studies involving MI [33,40–43,45,48,49,51,56–58,62]. In addition, there were 25 studies for Caucasians [23–25,27,30,38–45,47,48,50,51,53,56–58,60,62–64] and 19 studies for Asians [26,28,29,31–37,46,49,52,54,55,59,61,65,66]. A total of 28 studies with 23,959 subjects were included in the analysis [8,33,35,36,40,44,45,50,53,59,67–85]. Of these, there were 11 studies for Caucasians [8,40,44,45,50,53,67,69,71,81,83] and 17 studies for Asians [33,35,36,59,68,70,72–80,82,84,85].	5
**Data collection process**	10	Data were independently extracted from original publications by two reviewers (Minghong Yao and Yusong Ding) according to the inclusion criteria listed above. Discrepancy between the reviewers was resolved by consensus or a third reviewer (ShuXia Guo).	4
**Data items**	11	Data, including name of the first author, year of publication, study population (country, ethnicity), source of controls, case/control sample size, minor allele frequency (MAF), genotype counts in the cases/controls, and evidence of Hardy–Weinberg equilibrium (HWE), the population number, the mean of HDL-C level and its SD by genotypes, were extracted from each study.	4
**Risk of bias in individual**	12	The Newcastle-Ottawa Scale (NOS) was used to assessed the methodologic quality of the individual studies by two reviewers	4

**studies**		(Minghong Yao and Yizhong Yan) [16]. Each study was evaluated and scored based on three criteria: selection (4 stars), comparability (2 stars), and exposure (3 stars). The NOS point ranges between zero up to nine stars, and the studies with a score of equal to or higher than seven stars was considered to be of high quality. Any disagreement was resolved by discussion with a third reviewer (Jiaming Liu).	
**Summary measures**	13	The strength of associations between the CETP TaqIB polymorphism and atherosclerosis were assessed by summary odds ratios (ORs) with their 95% confidence intervals (CIs). A pooled standardized mean difference (SMD) and its 95% CIs were used for the meta-analysis of HDL-C level and the CETP TaqIB polymorphism	4
**Synthesis of results**	14	For relationship between the CETP TaqIB variant and AS, the combined ORs were respectively calculated for the allele contrasts (B1 allele vs. B2 allele), additive genetic model (*B1B1* vs. *B2B2*), recessive genetic model (*B1B1* vs. *B1B2* + *B2B2*) and dominant genetic model (*B1B1* + *B1B2* vs. *B2B2*), respectively. For relationship between CETP TaqIB variant and HDL-C, the combined SMD were respectively calculated for *B1B1* vs. *B2B2*, *B1B2* vs. *B2B2* and *B1B1* vs. *B1B2*. Heterogeneity across individual studies was calculated using the Cochran's -Q statistic and the I^2 statistic ($p < 0.10$ and $I^2 > 50\%$ indicated evidence of heterogeneity) [17,18]. With no heterogeneity among studies, the summary OR estimate of the each study was calculated by the fixed effect model (Mantel–Haenszel) [19]. Otherwise, the random effect model (DerSimonian and Laird) would be used [20,21]. Then the Galbraith plot and meta-regression were performed subsequently to explore the heterogeneity sources [22].	4
**Section/Topic**	**#**	**Checklist Item**	**Reported on page #**
**Risk of bias across studies**	15	An estimate of potential publication bias was carried out by Begg's funnel plot and Egger's regression test ($p < 0.05$ was considered representative of statistically significant publication bias)	4
**Additional analyses**	16	For the AS, Subgroup analyses were performed based on ethnicity (Caucasians and Asians), atherosclerotic disease (CAD, MI and IS), source of controls (population-based studies and hospital-based studies) and study type (case control study and cohort study); for the HDL-C, Subgroup analyses were performed based on ethnicity (Caucasians and Asians). Sensitivity analyses were performed by limiting the meta-analysis to studies conforming to HWE and sample size. the Galbraith plot and meta-regression were performed subsequently to explore the heterogeneity sources [22].	4
**RESULTS**			
**Study selection**	17	The flow diagram of the study selection for this meta-analysis was shown in the Figure 1. 44 studies with 20,866 cases and 21,298 controls were met the inclusion criteria and included to assess the association between the *CETP TaqIB* polymorphism and atherosclerosis [23–65]. Among these studies, there were 28 studies involving CAD [23–32,34–39,44,46,47,50,52–55,59–61,66] and 3 studies involving IS [63–65] and 12 studies involving MI [33,40–43,45,48,49,51,56–58,62]. In addition, there were 25 studies for Caucasians [23–25,27,30,38–45,47,48,50,51,53,56–58,60,62–64] and 19 studies for Asians [26,28,29,31–37,46,49,52,54,55,59,61,65,66]. A total of 28 studies with 23959 subjects were included in the analysis [8,33,35,36,40,44,45,50,53,59,67–85]. Of these, there were 11 studies for Caucasians [8,40,44,45,50,53,67,69,71,81,83] and 17 studies for Asians [33,35,36,59,68,70,72–80,82,84,85]	5, 16–19, 21
Study characteristics	18	Tables 1 and 2 shows the studies included in the meta-analysis and their main characteristics.	16–19
Risk of bias within studies	19	The NOS results were shown in Table 1 and Table2. The NOS results showed that the average scores were 6.8 and 6.4, respectively.	16–19
Results of individual studies	20	The main results of individual studies were shown in Figures 2 and 3 (Figures 2 and 3: Forest plots for the relationship between *CETP TaqIB* variant and AS risk and HDL-C).	22–23
Synthesis of results	21	For the association between the *CETP TaqIB* polymorphism and atherosclerosis risk, the results of all 33 comparisons showed evidence of significant association between the *CETP TaqIB* polymorphism and atherosclerosis, suggesting that the carriers of allele TaqIB-B1 had a higher risk of atherosclerosis than the non-carriers (OR = 1.15, 95% CI = 1.09–1.21) (Figure.1). Additive genetic model (*B1B1* vs. *B2B2*: OR = 1.26, 95% CI = 1.19–1.34), dominant genetic model (*B1B1* + *B1B2* vs. *B2B2*: OR = 1.20, 95% CI = 1.14–1.27) and recessive genetic model (*B1B1* vs. *B1B2* + *B2B2*: OR = 1.13, 95% CI = 1.08–1.18) were also included in the analysis of the association between the CETP TaqIB polymorphism and atherosclerosis risk and the results were similar with allele comparison (Figures S1–S3).<br>Figure 2. describes the result of the meta-analysis of HDL-C level and *CETP TaqIB* polymorphism and it strongly suggested that the carriers of	5-6, 20

		B1B1 genotype had lower level of HDL-C than those of B2B2 genotype (*B1B1* vs. *B2B2*: SMD = 0.50, 95% CI = 0.36–0.65). We also compared the carriers of B1B1 genotype with those of B1B2 genotype (Figure S4. *B1B1* vs. *B1B2*: SMD = 0.18, 95% CI = 0.10–0.26) and B1B2 genotype with those of B2B2 genotype (Supplementary Figure S5. *B1B2* vs. *B2B2*: SMD = 0.32, 95% CI = 0.21–0.42).	
Risk of bias across studies	22	Begg's funnel plot and Egger's regression test were performed to assess potential publication bias. For the CETP polymorphism and atherosclerosis risk (*B1* vs. *B2*), the shape of the funnel plot (Figure 3) did not reveal obvious asymmetry which means no publication bias. Then, it was confirmed by Egger's test ($p = 0.074$); For the CETP polymorphism and HDL-C (*B1B1* vs. *B2B2*), both of the shape of the funnel plot (Figure 4) and Egger's test ($p = 0.058$) did not reveal obvious asymmetry which means no publication bias.	7, 24
Additional analysis	23	Subgroup analyses: For the association between the *CETP TaqIB* polymorphism and atherosclerosis risk, Subgroup analyzes by ethnicity showed the significant associations in Asians (for B1 allele vs. B2 allele: OR = 1.24, 95% CI = 1.15–1.35, for *B1B1* vs. *B2B2*: OR = 1.52, 95% CI = 1.35–1.72, *B1B1* vs. *B1B2* + *B2B2*: OR = 1.41, 95% CI = 1.29–1.53, *B1B1* + *B1B2* vs. *B2B2*: OR = 1.28, 95% CI = 1.15–1.42) was consistent than that in Caucasians (for B1 allele vs. B2 allele: OR = 1.09, 95% CI = 1.04–1.16, for *B1B1* vs. *B2B2*: OR = 1.19, 95% CI = 1.11–1.27, for *B1B1* vs. *B1B2* + *B2B2*: OR = 1.05, 95% CI = 1.00–1.11, for B1B1+B1B2 VS B2B2: OR = 1.18, 95% CI = 1.11–1.25). In addition, significant associations were also found between this variant and the susceptibility to atherosclerosis in the population-based group (for B1 allele vs. B2 allele: OR = 1.11, 95% CI = 1.05–1.17, for B1B1 vs. B2B2: OR = 1.21, 95% CI = 1.13–1.29, for *B1B1* vs. *B1B2* + *B2B2*: OR = 1.09, 95% CI = 1.04–1.15, for *B1B1* + *B1B2* vs. *B2B2*: OR = 1.17, 95% CI = 1.10–1.25), hospital-based group (for B1 allele vs. B2 allele: OR = 1.20, 95% CI = 1.10–1.31, for *B1B1* vs. *B2B2*: OR = 1.42, 95% CI = 1.26–1.59, for *B1B1* vs. *B1B2* + *B2B2*: OR = 1.24, 95% CI = 1.14–1.35, for *B1B1* + *B1B2* vs. *B2B2*: OR = 1.28, 95% CI = 1.16–1.42), CAD group (for B1 allele vs. B2 allele: OR = 1.15, 95% CI = 1.08–1.24, for *B1B1* vs. *B2B2*: OR = 1.31, 95% CI = 1.21–1.43, for *B1B1* vs. *B1B2* + *B2B2*: OR = 1.19, 95% CI = 1.12–1.27), for *B1B1* + *B1B2* vs. *B2B2*: OR = 1.21, 95% CI = 1.13–1.31,), MI group (for B1 allele vs. B2 allele: OR = 1.10, 95% CI = 1.03–1.19, for B1B1 vs. B2B2: OR = 1.18, 95% CI = 1.08–1.29, for *B1B1* + *B1B2* vs. *B2B2*: OR = 1.17, 95% CI = 1.08–1.26), IS group (for B1 allele vs. B2 allele: OR = 1.39, 95% CI = 1.17–1.66, for *B1B1* vs. *B2B2*: OR = 1.92, 95% CI = 1.33–2.97, for *B1B1* vs. *B1B2* + *B2B2*: OR = 1.40, 95% CI = 1.09–1.79, for *B1B1* + *B1B2* vs. *B2B2*: OR = 1.76, 95% CI = 1.25–2.47), CCS group (for B1 allele vs. B2 allele: OR = 1.14, 95% CI = 1.10–1.18, for *B1B1* vs. *B2B2*: OR = 1.30, 95% CI = 1.21–1.39, for *B1B1* vs. *B1B2* + *B2B2*: OR = 1.16, 95% CI = 1.11–1.22, for *B1B1* + *B1B2* vs. *B2B2*: OR = 1.22, 95% CI = 1.15–1.30), CS group (for B1 allele vs. B2 allele: OR = 1.07, 95% CI = 1.01–1.13, for *B1B1* vs. *B2B2*: OR = 1.16, 95% CI = 1.03–1.30, for *B1B1* + *B1B2* vs. *B2B2*: OR = 1.15, 95% CI = 1.04–1.28) respectively. The main results of meta–analysis were shown in Table 3.<br>For the association between the CETP TaqIB polymorphism and HDL–C, subgroup analyzes by ethnicity certified that the effect on HDL-C were all significant in Asians (*B1B1* vs. *B2B2*: SMD = 0.43, 95%CI = 0.26–0.60; *B1B1* vs. *B2B2*: SMD = 0.14, 95% CI = 0.06–0.21; *B1B1* vs. *B2B2*: SMD = 0.35, 95% CI = 0.20–0.50) and Caucasians (*B1B1* vs. *B2B2*: SMD = 0.60, 95% CI = 0.37–0.83; *B1B1* vs. *B2B2*: SMD = 0.23, 95%CI = 0.10–0.35; B1B2 vs. B2B2: SMD = 0.29, 95% CI = 0.13–0.45) (Figure 2, Figures S4 and S5).<br>Sensitivity analyses: Sensitivity analysis was performed to determine whether the robustness of the study results. The included studies were limited to those conforming to HWE and sample size. Overall, the corresponding pooled ORs and SMD were not materially altered, either for the *CETP TaqIB* polymorphism and atherosclerosis risk or *CETP TaqIB* polymorphism and HDL-C. Results of the sensitivity analysis suggested that the overall results of this meta-analysis were relatively robust and credible. The main results of sensitivity analyses were shown in Table 3 and Figure S6–S11)<br>Heterogeneity analysis: For the relationship between the *CETP TaqIB* polymorphism and atherosclerosis, significant heterogeneity among the available studies were observed in the overall comparisons (for allelic model: $P_Q < 0.001$, $I^2 = 57.8\%$; for additive model: $P_Q < 0.001$, $I^2 = 55.8\%$; for recessive model: $P_Q < 0.001$, $I^2 = 52.0\%$; for dominant model: $P_Q = 0.001$, $I^2 = 41.7\%$). To clarify the sources of heterogeneity, we conducted the meta-regression analysis. The results shown that heterogeneity can be explained by the source of controls (for allelic model: $p = 0.046$, for additive model: $p = 0.025$, for dominant model: $p = 0.039$) and ethnicity (for additive model: $p = 0.048$).<br>For the relationship between the *CETP TaqIB* polymorphism and HDL-C level, significant heterogeneity among the available studies was also observed in the overall comparisons (for B1B1 vs. B2B2: $P_Q < 0.001$, $I^2 = 90.8\%$; for *B1B1* vs. *B1B2*: $P_Q < 0.001$, $I^2 = 79.9\%$; for *B1B2* vs. *B2B2*: $P_Q < 0.001$, $I^2 = 85.1\%$). Four studies were identified as the main contributors of heterogeneity for Asian studies [74,76,77,80] and four studies were identified as the main contributors of heterogeneity for Caucasian studies [44,50,67,69] using the Galbraith plot (Supplementary Figures S12 and S13). Figures S14–S16 shows that association between the *CETP TaqIB* polymorphism and HDL–C level after exclusion of these outlier studies. However, the significant association between the CETP polymorphism and HDL-C level was unchanged both in Asian subgroup (*B1B2* vs. *B2B2*: SMD = 0.47, 95% CI = 0.36–0.57; *B1B2* vs. *B2B2*: SMD = 0.19, 95% CI = 0.11–0.26; *B1B2* vs. *B2B2*: SMD = 0.28, 95% CI = 0.18–0.37) and Caucasian subgroup (*B1B2* vs. *B2B2*: SMD = 0.35, 95% CI = 0.30–0.40; *B1B2* vs. *B2B2*: SMD = 0.16, 95% CI = 0.12–0.19; *B1B2* vs. *B2B2*: SMD = 0.19, 95%CI = 0.15–0.20).	5–7, 20

**DISCUSSION**			
Summary of evidence	24	In the present meta-analysis, a total of 45 studies from 44 papers with 20,866 cases and 21,298 controls, we found that the TaqIB-B2 allele was significantly associated with reduced of atherosclerosis both in Caucasians and Asians. Additionally, 28 studies with 23,959 subjects were included in the analysis of association between the *CETP TaqIB* polymorphism and HDL-C level. According to the results, the TaqIB-B2 allele was significantly associated with a higher level of HDL-C both in Caucasians and Asians. Therefore, it is reasonable to assume that the *CETP TaqIB* polymorphism is probably by influencing the HDL-C metabolism to play protective factors for the development of atherosclerosis.	7
Limitations	25	**Limitations:** There are several potential limitations in our present meta-analysis should be acknowledged. Firstly, there was significant heterogeneity in our study. Although we have used appropriate meta-analytic techniques, we cannot completely exclude the influence of the heterogeneity. Secondly, it might miss the eligible articles that reported in other languages because our study only focused on articles published in English and Chinese languages. Thirdly, the sample sizes of some studies were rather small. In summary, it is well known that AS was affected by multiple environmental and genetic factors, we are only discussed a single gene polymorphism and not analyzed environmental factors, there are still many unclearly environmental and genetic factors and their interactions. Thus, it remains to be detected.	8
Conclusions	26	**Conclusion:** In conclusion, the present meta-analysis shows that the CETP TaqIB-B2 allele is associated with a higher serum HDL-C level and a protective role for AS risk both in Asians and Caucasians. Further investigations with the consideration of gene-gene and gene-environment interactions are needed.	8
**FUNDING**			
Funding	27	This work was supported by National Science and Technology Support Projects for the "Eleventh Five-Years Plan" of China (No. 2009BAI82B04), National Natural Science Foundation of China (No. 81560551) and Special Fund for Investigation of chronic heart and lung disease in Tibet and Xinjiang of China (No. 201402002).	Online submission system
